# APOE-high myeloid cells are uniquely associated with metastatic intrathoracic lymph nodes obtained by EBUS-TBNA in primary lung cancer

**DOI:** 10.1038/s41698-025-01091-5

**Published:** 2025-08-20

**Authors:** Seung-jae Kim, Minhyung Kim, Ki-Hyun Kim, Jacob D. Eccles, Bumseo Baek, Catherine Dell, Kevin Haas, Christopher M. Kapp, Christian Ascoli, Sungyong You, Gye Young Park

**Affiliations:** 1 Division of Pulmonary, Critical Care, Sleep and Allergy, Department of Medicine, University of Illinois at Chicago, Chicago, IL USA; 2 Departments of Urology and Computational Biomedicine, Cedars-Sinai Medical Center, Los Angeles, CA USA; 3 Jesse Brown Veterans Affairs Medical Center, Chicago, IL USA; 4 Present Address: Division of Pulmonary and Critical Care, Department of Medicine, Northwestern University, Chicago, IL USA

**Keywords:** Lung cancer, Metastasis, Tumour immunology, Diagnostic markers

## Abstract

The draining lymph node (LN) is the most frequent and often first site of cancer metastasis. Although endobronchial ultrasound-guided transbronchial needle aspiration (EBUS-TBNA) is frequently performed as a standard practice in lung cancer diagnosis and staging, its diagnostic accuracy remains modest, primarily due to the minuscule sample size of needle aspirates. With the advent of single-cell technologies, we comprehensively analyzed the immune cell repertoire in a series of EBUS-TBNA samples. Intrathoracic LN samples from 18 subjects with pathologically confirmed metastasis and four controls without evidence of metastasis were compared using single-cell RNA sequencing and mass cytometry analyses. We found that immune cell composition and gene expression patterns differed markedly between metastatic and control LNs. In particular, metastatic LNs contained relatively more APOE-high myeloid cells, with the latter exhibiting significant transcriptional derangement and a powerful intercellular interaction signature. Additionally, CD8 T cells in metastatic LNs demonstrated a unique exhausted phenotype. In conclusion, immune cell phenotypes and gene expression patterns from EBUS-TBNA samples can be leveraged to advance our understanding of cancer immunology and may have independent diagnostic value when malignant cells fail to be identified on histopathology.

## Introduction

Endobronchial ultrasound-guided transbronchial needle aspiration (EBUS-TBNA) is a minimally invasive procedure that enables access to intrathoracic lymph nodes (LNs) for diagnosis and staging of lung cancer. EBUS-TBNA has revolutionized lung cancer workup by improving mediastinal staging accuracy and minimizing the need for higher-risk surgical procedures^1–3^. As a result, EBUS-TBNA is currently recommended over surgical procedures as the preferred initial test for lung cancer staging due to its superior safety profile and cost-effectiveness^4,5^. However, the accuracy of EBUS-TBNA remains in modest ranges due to the high ratio of nondiagnostic samples, which is primarily due to the minuscule sample size of the needle aspirates^6^. Beyond the cytologic analysis for the presence or absence of malignant cells, there is a clear need to improve diagnostic yield by adopting innovative approaches. Here, we investigate the immune cell repertoire in EBUS needle aspirates and seek to identify associated features that relate to malignant metastasis.

Beyond its ability to identify the presence or absence of malignant cells within an intrathoracic LN, EBUS-TBNA offers invaluable access to immune cells within the cortex and medulla of these secondary lymphoid organs. While an extensive body of research has focused on immune cells within primary tumors, the immune landscape of intrathoracic LNs, particularly as it is altered by metastasis, remains underexplored. Notwithstanding, it is well recognized that metastatic cancer induces morphological changes in LNs, including ablation of the central hilar structure and overall enlargement, which can be appreciated by using EBUS ultrasound^7–9^. However, the phenotypic characteristics of immune cells associated with metastatic LNs remain largely unknown.

The tumor microenvironment (TME), whether primary or metastatic, is a complex but highly structured ecosystem comprising cancer cells and various non-malignant cells, including immune cells. Cancer cells actively recruit and reprogram host cells to create a supportive environment for their survival and proliferation^10^. Notably, current evidence suggests that cancer cells are also capable of manipulating and reprogramming immune cells within LNs to foster an environment conducive to metastasis. Although the exact mechanisms have yet to be fully elucidated, it is thought that cancer cells employ a multitude of intercellular crosstalk mechanisms that operate within the TME, including direct cell-to-cell contact and indirect paracrine signaling through cytokines, growth factors, and extracellular vesicles to achieve environmental remodeling^11^.

The advent of single-cell technologies and advances in the field have made it possible to conduct comprehensive analyses of immune cells acquired from fine needle aspirates. Leveraging single-cell technologies, we investigated the immune alterations in intrathoracic LNs harboring lung cancer metastases to gain deeper insights into the mechanisms of metastasis and to identify potential immune signatures associated with metastatic LNs.

## Results

### Characteristics of study subjects

All study subjects were individuals who underwent standard diagnostic bronchoscopy with EBUS to evaluate radiological abnormalities. At the time of enrollment, none of the subjects had received any anti-cancer treatment. The enrolled study subjects were divided into two groups: the non-metastatic control group (control, *n* = 4) and the group with pathologically proven primary lung malignancy and evidence of metastasis within the intrathoracic LNs (metastatic LN, *n* = 18). The control group consisted of adult subjects aged 60 to 80 years (median age: 66.5). The metastatic LN group included adult subjects aged 50 to 80 years (median age: 67.5), with diverse self-reported racial and ethnic backgrounds: African American (*n* = 10), Caucasian (*n* = 5), Hispanic (*n* = 2), and Asian (*n* = 1). All study subjects were either former or active smokers with a median of 27.5 and 40 pack-year history in the metastatic LN and control groups, respectively. There was no significant difference observed between groups regarding age and smoking history (data not shown, Mann-Whitney test, p value > 0.05). Pathologies of the metastatic LNs were adenocarcinoma (*n* = 10), squamous cell carcinoma (*n* = 4), small cell lung cancer (*n* = 2), non-small cell carcinoma (*n* = 1), and poorly differentiated carcinoma (*n* = 1). Each sample was individually processed for scRNA-seq. Total captured cell counts, remaining cell counts after filtering out low-quality cells, and immune cell counts are listed in Table 1.

**Table 1:** Sample information and cell counts detected in scRNA-seq analysis

Categories	ID	Age Range	Race	Smoking history		Pathology	Cell count for scRNAseq		
				Status	Pk-yr		Total	Remaining	Immune
Control LN Group	N1	60–70	Black, Not Hispanic or Latino	Former	50		9600	8513	6386
	N2	60–70	White, Not Hispanic or Latino	Active	40		8599	7346	5814
	N3	60–70	Black, Not Hispanic or Latino	Former	40		22,805	11,622	9827
	N4	70–80	White, Hispanic or Latino	Active	22		7358	6482	5927
Metastatic LN Group	P1	70–80	Black, Not Hispanic or Latino	Active	25	Adenocarcinoma	17,378	14,963	4877
	P2	60–70	Asian	Former	1	Adenocarcinoma	25,777	22,770	44
	P3	60–70	White, Not Hispanic or Latino	Former	22.5	Adenocarcinoma	13,615	6124	2775
	P4	70–80	Black, Not Hispanic or Latino	Active	50	Adenocarcinoma	26,727	23,859	10,217
	P5	70–80	Black, Not Hispanic or Latino	Active	50	Adenocarcinoma	9408	8825	466
	P6	70–80	Black, Not Hispanic or Latino	Active	25	Adenocarcinoma	21,951	9093	7557
	P7	60–70	Black, Not Hispanic or Latino	Former	50	Adenocarcinoma	1988	1796	441
	P8	60–70	White, Not Hispanic or Latino	Active	23	Adenocarcinoma	8915	4380	3624
	P9	50–60	White, Not Hispanic or Latino	Former	21	Adenocarcinoma	15,089	8786	5123
	P10	70–80	Black, Not Hispanic or Latino	Former	30	Adenocarcinoma	23,325	12,253	3253
	P11	70–80	Black, Not Hispanic or Latino	Active	45	Squamous cell carcinoma	2789	1845	449
	P12	50–60	White, Not Hispanic or Latino	Active	120	Squamous cell carcinoma	7560	6358	3477
	P13	60–70	White, Hispanic or Latino	Former	50	Small cell lung cancer	19,352	17,586	3706
	P14	50–60	Black, Not Hispanic or Latino	Former	13	Squamous cell carcinoma	6014	4507	3809
	P15	70–80	Black, Not Hispanic or Latino	Active	45	Small cell carcinoma	12,266	11,074	914
	P16	50–60	Black, Not Hispanic or Latino	Active	23	Squamous cell carcinoma	1541	551	19
	P17	60–70	White, Not Hispanic or Latino	Former	42	Non-small cell carcinoma	17,271	10,492	4425
	P18	70–80	White, Hispanic or Latino	Former	4	Poorly differentiated carcinoma	2161	1481	941

### Identification of immune cell types in EBUS-TBNA using scRNA-seq and CyTOF

**Fig. 1:**
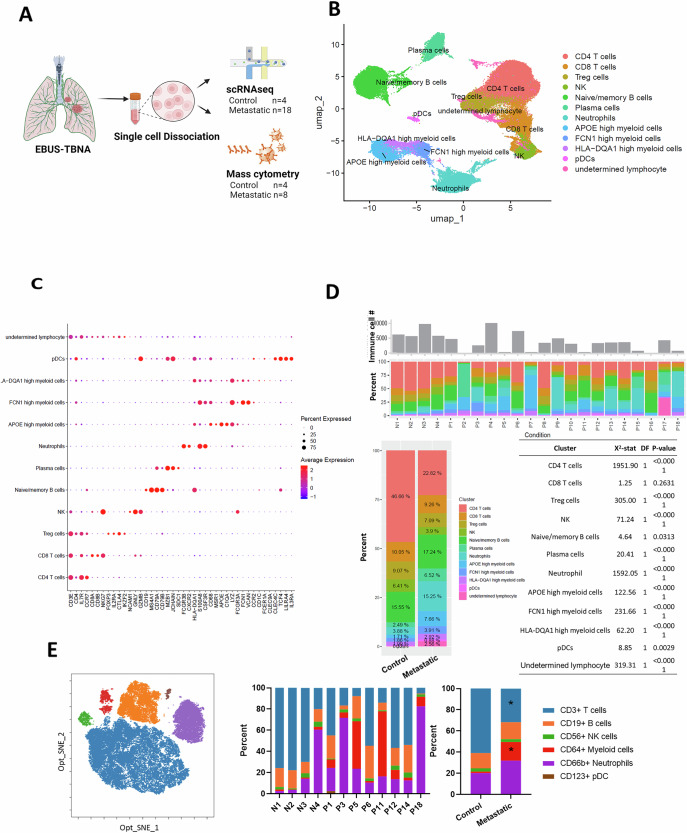
Identification of immune cell types in EBUS-TBNA using scRNA-seq and CyTOF. **A** Schematic representation of the study design, illustrating the collection of EBUS-TBNA samples for scRNA-seq (*n* = 4 controls and *n* = 18 metastatic LN) and for mass cytometry (CyTOF) analyses (*n* = 4 controls and *n* = 8 metastatic LN). **B** UMAP plot demonstrating the 1^st^ and 2^nd^ component of pooled scRNA-seq data from both control and metastatic LN groups identifying immune cell clusters. **C** Dot plot showing the expression of canonical marker genes for immune cell types. **D** Bar plot demonstrating the number of immune cells used for analysis and stacked bar plot depicting the relative proportions of major immune cell types identified in individual control (N1–N4) and metastatic LN (P1–P18) samples. The percentage and Chi-squared test result of each cell type in control and metastatic LN groups are shown. Cell types are color-coded as indicated in the legend. **E** Opt_SNE plot of CyTOF data showing clusters of pooled immune cells from control and metastatic LN groups on the 1st and 2nd components and stacked bar plot depicting the percentage of individual samples and the per group mean for major immune cell types. Statistical significance was determined by the Mann–Whitney test (*p < 0.05).

Non-immune cells, such as cancer cells and structural cells, were excluded from the dataset in silico to analyze the immune cell composition of EBUS needle aspirates (see Materials and Methods). The scRNA-seq data were pooled for immune cell annotation. Dimensionality reduction using the UMAP demonstrated twelve distinct immune cell clusters based on their transcriptomic profiles (Figs. 1B and S1A). All but one cluster (undetermined lymphocytes) exhibited characteristic canonical cell type-specific gene expression patterns: CD4 T cells (*CD3E, CD4*), CD8 T cells (*CD3E, CD8A*), Treg cells (*CD3E, CD4, FOXP3, IL2RA*), NK cells (*NKG7, NCAM1*), naïve/memory B cells (*CD19, MS4A1*), plasma cells (*CD79A, MZB1*), neutrophils (*S100A8, CSF3R*), plasmacytoid DC (pDC) (*TCF4, LILRA4, IL3RA*) and three myeloid cell clusters: APOE-high myeloid cells (*CD68, APOE, C1QA*), FCN1-high myeloid cells (*CD68, FCN1, VCAN*), and HLA-DQA1-high myeloid cells (*HLA-DQA1, CD1C, CLEC9A*). The expression patterns of canonical immune cell marker genes were consistent with the previously reported immune cell annotation database (Fig. 1C)^12^.

Next, we quantitatively analyzed the relative abundance of each immune cell cluster in control versus metastatic lymph nodes using scRNA-seq data (Fig. 1D and Table S1). The analysis revealed significant differences in immune cell distributions between the two groups. In control lymph nodes, CD4 T cells were the most abundant population, comprising 46.66% of total immune cells, followed by naïve/memory B cells (15.55%) and CD8 T cells (10.05%). In contrast, in metastatic lymph nodes, there was a marked reduction in CD4 T cell abundance to 22.82% (*p* < 0.01, Chi-squared test) and a relative increase in neutrophils from 3.88% to 15.25% (*p* < 0.01) and APOE-high myeloid cells from 1.71% to 7.66% (*p* < 0.01) (Fig. 1D and Table S1).

CyTOF analysis corroborated the scRNA-seq observation of increased myeloid representation in metastatic LNs (Fig. 1E and Fig. S1B). Since FCGR1A (encoding CD64), a broadly expressed myeloid marker, is detected in both APOE-high and FCN1-high subsets by scRNA-seq (Fig. S1C), the elevated CD64 signal from mass cytometry likely reflects the combined expansion of these populations within the metastatic microenvironment.

Next, we assessed inter-individual variability by comparing cell cluster percentages from individual patients within control and metastatic groups. scRNA-seq analysis revealed significant shifts in the metastatic group, with a notable decrease in CD4 T cells and an increase in APOE-high myeloid cells (Mann-Whitney test, Fig S2A). Concordantly, CyTOF data showed similar trends: CD3 + T cells decreased, while CD64+ myeloid cells increased (Mann-Whitney test, Fig S2B).

### Distinct myeloid cell clusters in EBUS-TBNA samples

**Fig. 2:**
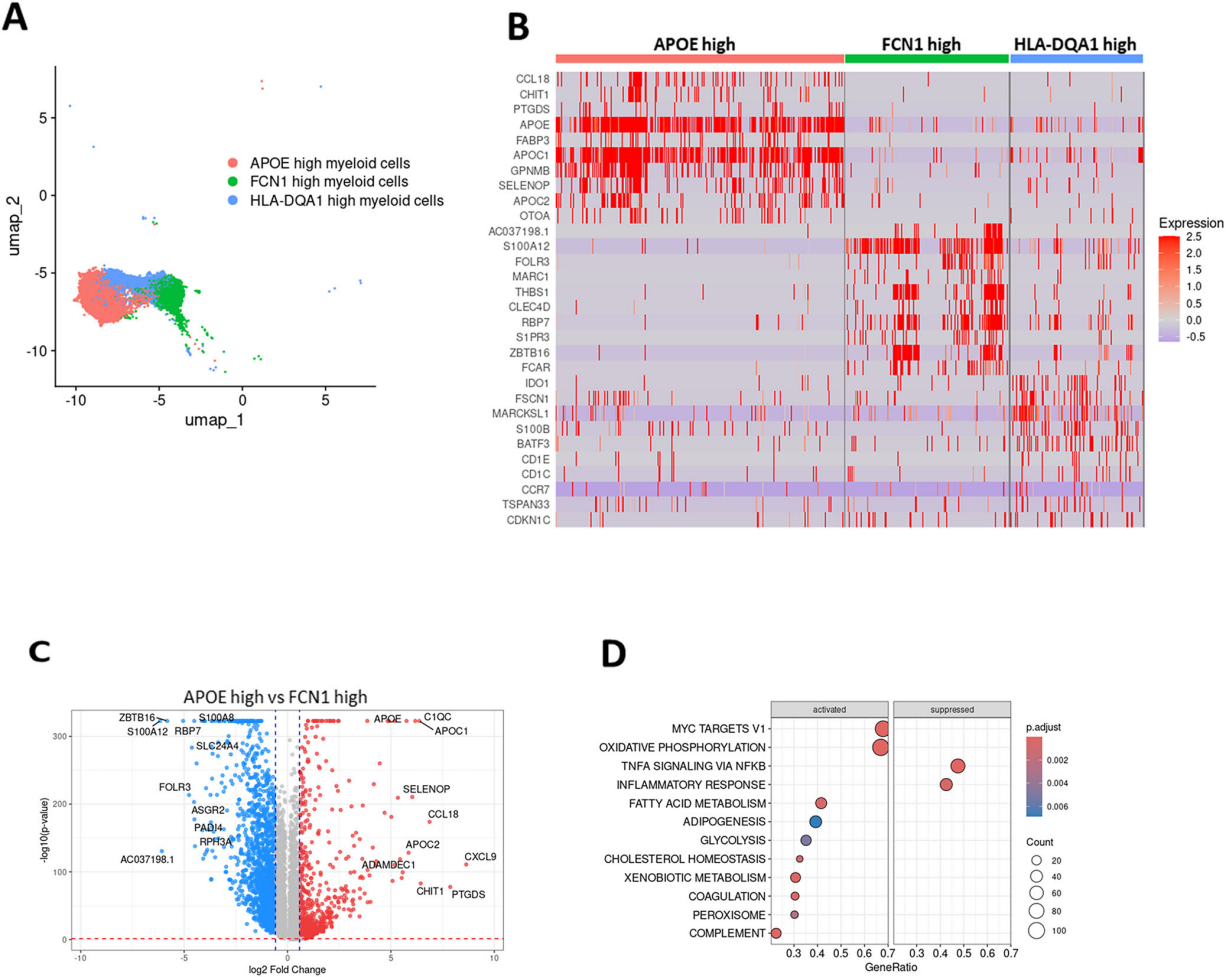
Distinct myeloid cell populations in EBUS-TBNA Samples. **A** UMAP plot demonstrating the pooled scRNA-seq data identifying myeloid cell clusters. **B** Heatmap displaying the expression of top 10 genes of each myeloid subtypes. **C** Volcano plot illustrating differential gene expression between APOE-high and FCN1 high myeloid cells. **D** Gene set enrichment analysis of APOE-high myeloid cells as compared to FCN1 high myeloid cells.

We identified three distinct myeloid cell clusters in the intrathoracic LNs based on the scRNA-seq data (Fig. 1). To further characterize these clusters (Figs. 1B and 2A), we compared their gene expression profiles and functional pathways (Fig. 2). HLA-DQA1-high myeloid cells exhibited high expression of DC markers, including BATF3, CD1C, CLEC9A, and IDO1, confirming their DC identity (Fig. 2B)^13,14^.However, APOE-high and FCN1-high myeloid cell clusters express common macrophage markers such as FCGR1A (CD64), indicating macrophage identity (Fig. S1C).

We compared these two macrophage clusters: APOE-high and FCN1-high myeloid cells (Fig. 2B–D). APOE-high myeloid cells express *APOC1, SELENOP* and *C1QA*, corresponding to tissue-resident macrophages found in TME^15,16^, Gene Set Enrichment Analysis (GSEA) display immunosuppressive traits, characterized by elevated M2 phenotype markers, OXPHOS pathway genes, and CCL18, alongside suppressed inflammatory response and TNFα signaling (Fig. 2D)^17^. In contrast, FCN1-high myeloid cells displayed an M1-like macrophage profile, with upregulation of pro-inflammatory genes (*S100A8, S100A12, CXCL8, CCL3*) and enrichment of the pathway genes for TNF-α signaling via NFκB^18,19^.

### Highest transcriptional alteration observed in metastatic APOE-high myeloid cells

**Fig. 3:**
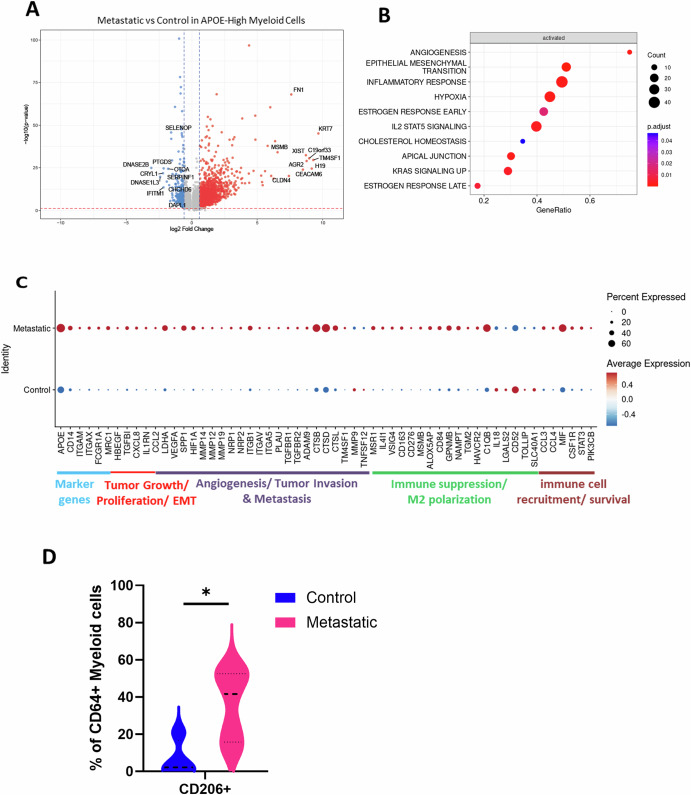
APOE-high myeloid cells exhibit a pro-tumoral phenotype in metastatic LNs. **A** Volcano plot illustrating differential gene expression in APOE-high myeloid cells between metastatic LN and control LN groups. **B** Enriched APOE-high myeloid cell pathways in the metastatic LN group as compared to controls. **C** Dot plot categorized by functional groups in APOE-high myeloid cells showing the differential expression of select genes between control and metastatic LN groups. **D** Violin plot demonstrating percentages of CD206 + CD64+ myeloid cells between control and metastatic LN groups from the CyTOF data. Statistical significance was determined by Mann–Whitney test (*p < 0.05).

**Fig. 4:**
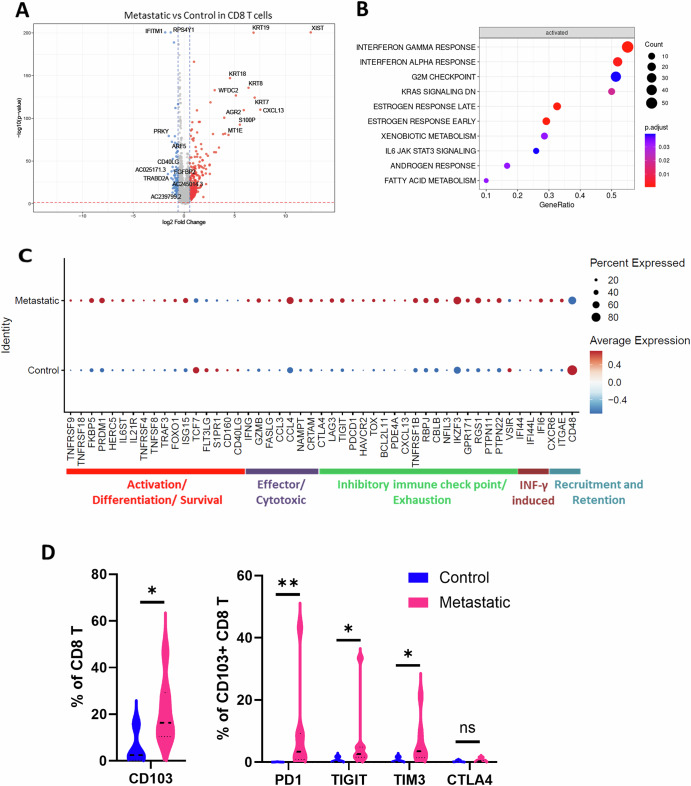
Metastatic LNs are occupied by unique CD8 T cell phenotypes. **A** Volcano plot illustrating differential gene expression in CD8 T cells between metastatic LN and control LN groups. **B** Enriched CD8 T cell pathways in the metastatic LN group as compared to controls. **C** Dot plot categorized by functional groups in CD8 T cells showing the differential expression of select genes between control and metastatic LN groups. **D** Violin plot demonstrating percentages of CD103 (ITGAE) positive CD8 T cells between control and metastatic LN groups from CyTOF data. Additionally, the percentage of PD1, TIGIT, TIM3 and CTLA4 positive cells among CD103 + CD8 T cells was compared between the two groups. Statistical significance was determined by Mann-Whitney test (**p < 0.01, *p < 0.05).

In addition to the differences in the relative abundance of immune cells (Fig. 1D), we further analyzed differences among immune cell clusters between the control and metastatic LN groups by comparing the gene expression patterns. For each immune cell cluster, we assessed DEGs and performed biological pathway enrichment analysis to characterize the function of DEGs as described in Methods. Furthermore, we categorized the functional genes related to the TME among the DEGs. These comparative analyses were performed in CD4 T cells (Fig. S5), CD8 T cells (Fig.4), Treg cells (Fig. S6), NK cells (Fig. S7), naïve/memory B cells (Fig. S8), plasma cells (Fig. S9), neutrophils (Fig. S10), pDC (Fig. S13) and three myeloid cell clusters: APOE-high myeloid cells (Fig. 3), FCN1-high myeloid cells (Fig. S11), and HLA-DQA1-high myeloid cells (Fig. S12). We found that the patterns of immune cell gene expression were significantly different between the control and metastatic LN groups (Fig. S3). Of note, APOE-high myeloid cells exhibited the largest number of DEGs, indicating that metastasis of malignant cells into LNs either induces substantial transcriptional alteration in resident cells or brings new migratory populations to the LN. In contrast, the gene expression patterns of pDCs were relatively unaffected by metastasis, showing the smallest number of DEGs (Fig. S3).

### APOE-high myeloid cells exhibit a pro-tumoral phenotype in metastatic LNs

Myeloid cells, including monocytes, macrophages, and DCs, are major components of the TME, playing multifaceted functional roles through their potent immune regulatory or immune promoting capacities. Our data showed that the proportion of APOE-high myeloid cells significantly increased in metastatic LNs (Fig. 1D and Table S1). In particular, this cluster exhibited the highest number of DEGs among immune cells in the metastatic LN group compared to controls (Fig. S3). Upon comparison of gene expression patterns between APOE-high myeloid cells in the control and metastatic LN groups, we found that the biological pathways related to angiogenesis, epithelial-mesenchymal transition (EMT), inflammatory response, and hypoxia were enriched in the APOE-high myeloid cells of the metastatic LN group (Fig. 3B). This data implies the potential role of APOE-high myeloid cells in establishing a metastatic milieu. Notably, among DEGs, specific genes associated with tumor growth and EMT (*HBEGF, TGFBI, CXCL8*)^20,21^, angiogenesis and tumor invasion (*VEGFA, SPP1, MMPs*)^22^, immune suppression/M2 polarization (*MRC1, MSR1, CD163*)^23^, and immune cell recruitment/survival (*MIF, CSF1R*)^24^ were upregulated in metastatic LN group (Fig. 3C). These gene expression profiles suggest that APOE-high myeloid cells in metastatic LNs exhibit characteristics of tumor associated macrophages (TAMs) with pro-tumor properties^25–27^ and this finding is consistent with recently reported data showing the immunosuppressive role of APOE-expressing macrophages existing in primary tumor mass of gastric cancers, melanoma, and other types of cancer^27–29^. Interestingly, within the top 20 DEGs (Fig S4), genes such as *KRT7, FN1, CD276*, and *CLEC5A* exhibited highly specific expression in metastatic APOE-high myeloid cells. These genes may represent potential diagnostic markers for the identification of metastatic APOE-high myeloid cells in EBUS-TBNA samples, pending further validation. Additionally, our CyTOF data showed a significant increase in CD206 + CD64+ myeloid cells in the metastatic LN group, although our CyTOF data was limited by the small number of myeloid markers (Fig. 3D). CD206, encoded by *MRC1*, is a well-established marker for M2-polarized macrophages, functionally associated with pro-tumorigenic TME^23^. Collectively, these results suggest that APOE-high myeloid cells in metastatic LNs highly express the genes supporting tumor growth, angiogenesis, and immune evasion.

### Metastatic LNs are occupied by unique CD8 T cell phenotypes

Despite comparable relative abundance between groups, CD8 T cells play a critical role in anti-tumor cytotoxicity, and promulgation of CD8 T cell exhaustion is one of the mechanisms by which cancer cells evade anti-tumor immunity. Therapeutic agents aimed at reestablishing CD8 T cell function have been established as standard anti-cancer immunotherapy. To investigate how metastatic LNs alter CD8 T cell transcriptome, we compared their gene expression patterns between the control and metastatic LN groups (Fig. 4A), although there was no significant difference in the population size between the two groups (Figs.1D and S2A). We identified *XIST, KRT19, KRT18, KRT8, KRT7, WFDC2, AGR2, CXCL13, S100P*, and *MT1E* as the top 10 upregulated genes by comparing CD8 T cells from metastatic and control LN groups (Fig. 4A). In particular, CD8 T cells that express high levels of keratins, *S100P* and *CXCL13*, are associated with immunosuppression and poor prognosis^30–33^. Further assessment by GSEA also revealed that CD8 T cells in the metastatic LN group have significant enrichment of genes associated with interferon (IFNγ and IFNα) pathways and checkpoint genes (Fig. 4B), suggesting an active modulation of the CD8 T functions in the interferon-rich microenvironment^34,35^. Additionally, within the TME of the metastatic LN group, there was significant upregulation of genes related to CD8 T cell functions including cellular activation and differentiation (*TNFRSF18, PRDM1*)^36^, cytotoxic effector function (*IFNG, GZMB*)^37^, and immune cell recruitment and retention (*ITGAE, CXCR6*)^38^, suggesting greater cytotoxic response and retention. Paradoxically, these CD8 T cells simultaneously expressed higher inhibitory immune checkpoint and exhausted phenotype genes (*PDCD1, LAG3*)^39^ (Fig. 4C). Consistent with the transcriptomic results, the CyTOF demonstrated that CD103+ (*ITGAE*) CD8 T cells were significantly increased in metastatic LNs. These cells also exhibit higher expression of inhibitory receptors PD1(*PDCD1*), *TIGIT*, and TIM3 (*HAVCR2*), consistent with the reported exhausted tissue-resident memory CD8 T cell phenotype^38^ (Fig. 4D). Although we could not further dissect CD8 T cells into sub-clusters because of the limited cell numbers in the EBUS samples, the co-existence of cytotoxic effector and exhausted transcriptomic programs in CD8 T cells suggests that intrathoracic LNs in metastatic disease harbor a heterogeneous population of CD8 T cell subsets with specific phenotypes that favor cancer progression.

### APOE-high myeloid cells and CD8 T cells exhibit enhanced cell–cell interactions in metastatic LNs

**Fig. 5:**
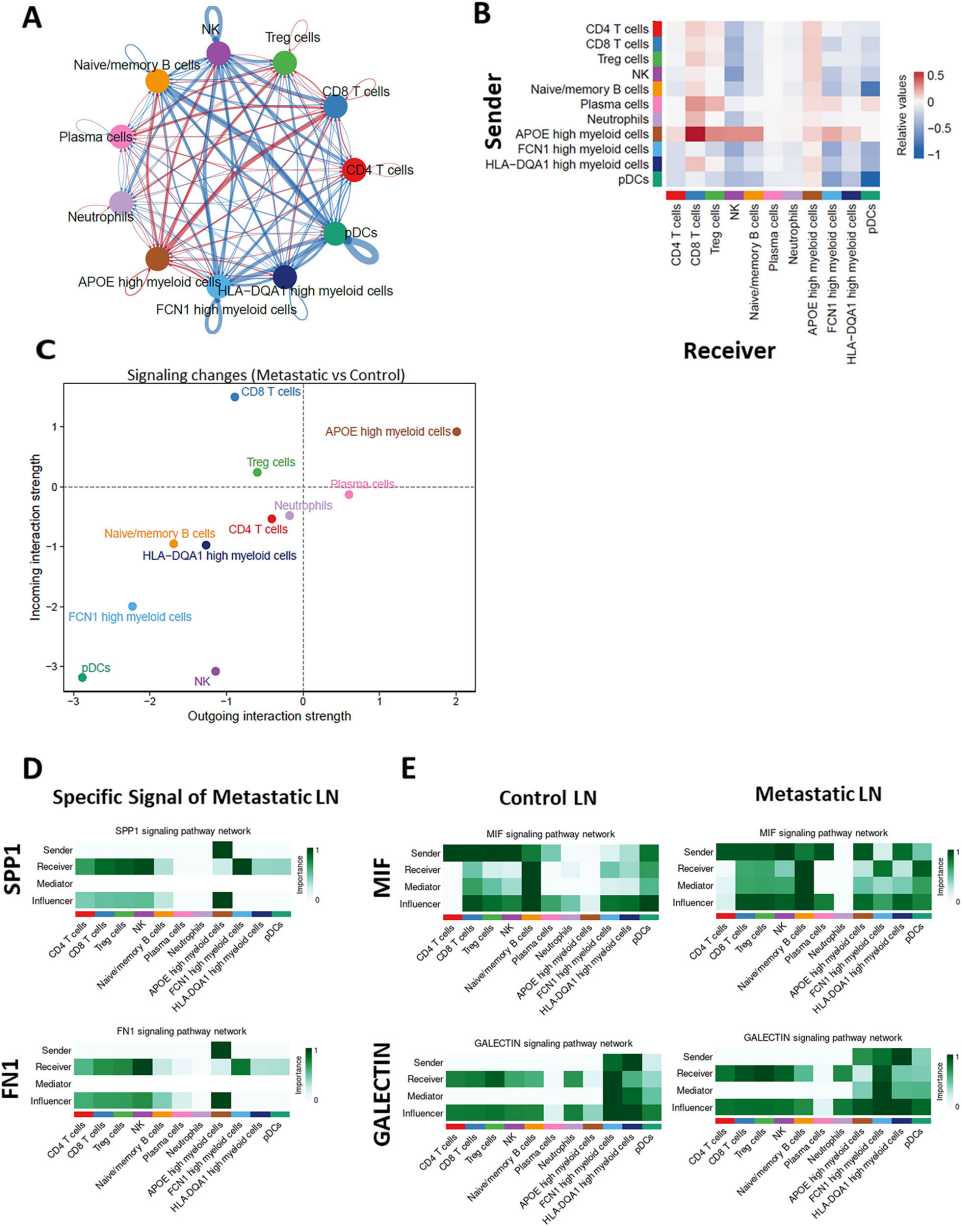
APOE-high myeloid cells and CD8 T cells exhibit enhanced cell-cell interactions in metastatic LNs. **A** A network model illustrating the cell-cell interactions among immune cell types and the differential interaction strength between control LN and metastatic LN groups. The nodes and edges represent the immune cell types and the interactions between the cell types, respectively. Red and blue edges represent upregulated and downregulated interactions, respectively, in the metastatic LN group compared to control LN group. The thickness of the edge is indicative of the strength of the interaction. **B** A heatmap depicting the relationship among immune cells. The rows represent outgoing interactions from the corresponding immune cell type, while the columns represent incoming interactions received by the corresponding immune cell type. The color gradient represents the strength of the interaction. The colors red and blue indicate and increase and decrease, respectively, in interaction strength in the metastatic LN group compared to control LN group. **C** Scatter plot showing changes in the strength of incoming and outgoing interactions for each cell type in metastatic LN compared to control LN groups. **D** Heatmaps displaying signal pathway networks of SPP1 and FN1 in the metastatic LN group. **E** Heatmaps displaying signal pathway networks of MIF and GALECTIN in the control and metastatic LN groups.

Immune cells communicate with each other via direct or indirect ways to generate unique TME in malignancy. To infer how immune cells communicate via ligand-receptor interactions or other soluble factors in the control and metastatic LN environments, we performed CellChat analysis as described in Methods. This unsupervised analysis enabled us to quantify the probability of cell-cell interactions by integrating scRNA-seq data with established databases of ligand-receptor interactions and their cofactors^40^. We assessed the strength and frequency of immune cell-cell interactions, excluding the influence of cancer or structural cells, in both control and metastatic LN groups (Fig. S14) and compared the differential interaction strengths between the groups (Fig. 5A). Compared to the control LNs, metastatic LNs manifest altered cellular interactions. Most notably, the interaction between APOE-high myeloid cells and CD8 T cells was strongly enhanced, whereas interactions originating from pDCs were diminished (Fig. 5A–C). APOE-high myeloid cells demonstrated a high degree of interaction with other cell types as signal senders (outward interactions), suggesting their role as primary ligand producers. In contrast, CD8 T cells exhibited a high degree of inward interactions, indicating their role as major signal receivers (Fig. 5A–C). Next, we further examined the details of cellular signaling pathways. Compared to the control LN group, the metastatic LN group uniquely displays enrichment of the SPP1 and FN1 signaling pathways with APOE-high myeloid cells serving as senders and other immune cells, such as CD8 T cells, CD4 T cells, Treg, and NK cells, serving as receivers (Fig. 5D). Even though some signaling pathways exist among immune cells in both control and metastatic LNs, they differed in the signal strength and manner of interaction between the groups. For example, the MIF and GALECTIN pathways are not well utilized by APOE-high myeloid cells in control LNs. However, APOE-high myeloid cells became actively engaged in cellular communication via these pathways in the metastatic LNs (Fig. 5E). Of interest, SPP1, FN1, MIF and GALECTIN signal pathways are all known to play a role in immunosuppression and pro-tumorigenic function within the TME^41–44^, suggesting a possible contribution to creating a lymphoid tissue microenvironment that faciliate metastatic infiltration.

### Validation of APOE-high myeloid cell enrichment in metastatic LNs using an independent cohort

Lastly, to validate our findings in an independent cohort, we utilized a scRNA-seq dataset in the public domain (GSE131907) obtained from patients with lung cancer^45^. The validation cohort was comprised of a mix of surgical resected control specimens and EBUS needle aspirates from patients with lung cancer^45^. To match our study’s criteria, we regrouped EBUS needle aspirates into confirmed metastatic LNs (*n* = 6) and control LNs (*n* = 10), which were surgically collected and proven to be cancer-free.

**Fig. 6:**
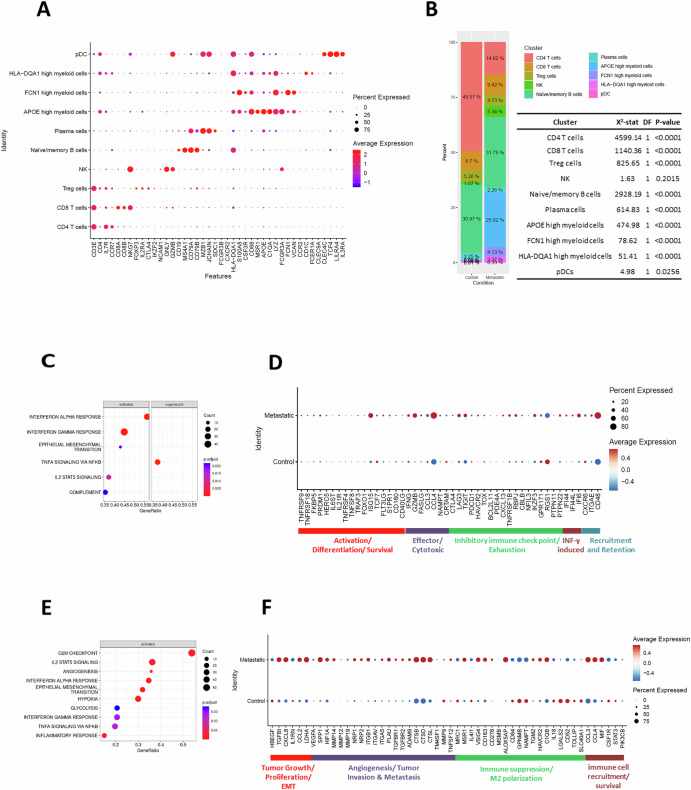
Validation of APOE-high myeloid cell enrichment in metastatic LNs using an independent cohort. **A** Dot plot showing the expression of canonical marker genes for immune cell types. **B** Stacked bar plots showing the percentage and Chi-squared test result of each cell type in the control and metastatic LN groups. Cell types are color-coded as indicated in the legend. **C** Enriched pathways in the metastatic LN group compared to control LN group in CD8 T cells. **D** Dot plot showing the differential expression of select DEGs categorized by functional groups in CD8 T cells from the control and metastatic LN groups. **E** Enriched pathways in the metastatic LN group compared to control LN group in APOE-high myeloid cells. **F** Dot plot showing the differential expression of select DEGs categorized by functional groups in APOE-high myeloid cells from the control and metastatic LN groups.

Based on gene expression pattern clustering, we identified cell types and cell clusters similar to those in our cohort (Figs. 1C and 6A). The relative abundance and distribution of immune cells in the validation cohort were, for the most part, comparable to our study (Figs. 1D, 6B, S15 and Table S2). The validation cohort had a significant increase in APOE-high myeloid cells in the metastatic LNs and significant reduction in CD4 T cells (Fig. 6B, S15 and Table S2). CD8 T cells in metastatic LNs had upregulated pathways associated with IFNγ and IFNα responses (Fig. 6C) and genes related to cell survival, cytotoxic effector functions, exhaustion, and tissue residency (Fig. 6D). Similarly, the data from APOE-high myeloid cells (Fig. 6E, F) in the validation cohort also supports our findings, showing enrichment of genes in pathways related to angiogenesis, EMT, and hypoxia. Despite differences in the study population and sampling methods, the overall patterns of immune cell alterations observed in metastatic LNs were consistent, particularly in CD8 T cells and APOE-high myeloid cells with our study.

## Discussion

Myeloid cells, known for their heterogeneity and plasticity, play a complex role in the TME, possessing the potential to either support or inhibit tumor growth and progression. We identified three distinct myeloid cell-types in both control and metastatic LNs that cluster in close proximity on the UMAP by gene expression. HLA-DQA1-high myeloid cells exhibited high expression of DC marker genes (Fig.2) with a potential role in antigen presentation and T cell activation within the lymph node environment. Next, FCN1-high myeloid cells displayed a gene expression profile consistent with M1-like macrophage polarization (Fig. 2). suggesting a role in inflammatory responses and immune cell recruitment. However, metastasis did not significantly alter their cell proportions (Fig. S2). Lastly, APOE-high myeloid cells showing immunosuppressive phenotype (Figs.2 and 3) are most active with respect to outgoing signals, notably expressing genes associated with tumor growth, EMT, angiogenesis, and immune suppression in metastatic TME (Figs. 3 and 5). These results align with the established multifaceted roles of APOE+ macrophages as TAMs in promoting tumor progression^25,26^. Among various tumor-enhancing pathways mediated by TAMs, the EMT pathway is one of the key mechanisms. TAMs secrete pro-EMT factors such as TGF-β and CCL18, which induce mesenchymal transition in neighboring tumor cells. This paracrine EMT induction facilitates tumor cell migration, invasion, and ultimately metastatic spread^26^. In parallel, CD8 T cell exhaustion appears to correlate with APOE-high myeloid cell predominance and signaling activity, lending support to the notion that APOE-high myeloid cells are TAMs and play a significant pro-tumorigenic role in metastatic LNs.

These two findings are of great interest given that APOE+ macrophages have been reported to exist and play an immunosuppressive role in the primary tumor mass of various types of cancer. However, their presence at sites of metastasis and their relevance in human disease have not been thoroughly evaluated. In pancreatic cancer, APOE+ macrophages inhibit CD8 T cell infiltration by producing CXCL1 and CXCL5^28^. Additionally, a recent pan-cancer analysis using data in the public domain revealed that APOE+ TAMs are associated with poor responses to immune checkpoint inhibitors due to their interaction with exhausted CD8 T cells^29^. Furthermore, in gastric cancer, APOE+ TAMs facilitate tumor cell migration by transferring apolipoprotein E via exosomes, leading to cytoskeletal remodeling in cancer cells^27^. Together, our data showing the high presence of APOE-high myeloid cells in metastatic intrathoracic LNs suggest that these cells may facilitate the establishment of metastatic niches by generating an immunosuppressive environment in the lymph nodes.

In our analysis, we observed the expression of cytokeratins (*KRT7, KRT8, KRT19*) in immune cell clusters from metastatic lymph nodes (Figs. 3A and 4A). While biologically unexpected, similar observations have been reported in other scRNA-seq datasets, including the Human Lung Cell Atlas^46^. Detection of these mRNAs does not necessarily indicate protein expression and may result from technical artifacts^47^. Notably, these transcripts persisted even after applying an alternative doublet filtering tool, DoubletFinder^48^. Therefore, the biological relevance of these observations remains unclear, and the findings should be interpreted with caution and validated through additional studies.

Though the immune cell landscape of metastatic LNs has been previously described in malignancies other than lung cancer^49–51^, our study provides novel insights into the utility of a combined approach with scRNA-seq and mass cytometry to evaluate minimally invasive EBUS-TBNA, highlighting its potential to characterize immune cell alterations in metastatic lymph nodes. The resulting data sheds light on characteristic changes, including an expansion of APOE-high myeloid cells and altered cellular programing of CD8 T cells, within the immunological networks at play within LN metastases, suggesting signaling pathways to be intervened on with future therapeutics. In particular, the interplay between APOE-high myeloid cells and other immune cells provides clues for understanding metastatic niche formation, TME immune modulation, and immunotherapy resistance. Additionally, and in an immediate sense, these data illustrate a myeloid signature that may serve as a cancer biomarker when insufficient biopsy tissue is negative or equivocal for malignant cells. In the future, combining multimodal sequencing (e.g., CITE-seq) with conventional methods such as multiplex immunofluorescence could identify robust protein markers that mirror the mRNA signatures, thereby improving the diagnostic yield of EBUS-TBNA in challenging cases.

It should be recognized that this study has weaknesses that limit the interpretation of the generated data which should be addressed in future studies. As primary matters, our study is limited by relatively broad inclusion criteria and small cohort size, as well as the sampling modality, EBUS-TBNA. Specifically, while EBUS-TBNA spares the morbidity of surgery and carries a low risk of bleeding, this approach provides a low volume of tissue and relatively low cellular yield. In doing so, it may fail to directly capture metastatic cancer cells within a given LN and thus may undersample the adjacent TME. Additionally, even if cancer cells are present in the sample, no spatial information is available with this modality, and tumor-infiltrating cells cannot be differentiated from peripheral bystanders. Furthermore, although the overall bleeding risk is low, any small amount of bleeding may contaminate the sample, again compounded by the low cell count of the desired tissue. Indeed, while collecting samples for the study, samples containing an excess of red blood cells were excluded, though a small amount of contamination was unavoidable. Additionally, the majority of our data was derived from scRNA-seq, which uses fewer cells than flow cytometry or bulk RNA-seq might accommodate. That being said, the quality and quantity of data obtained on a per cell basis with scRNA-seq is unparalleled. Lastly, this study did not account for details of the primary tumor, which likely influences the behavior of its metastases. Primary tumors were not sampled, and we cannot speak to the TME at those sites in any manner. Tumor biology was also outside the scope of this study, wherein incredible diversity exists in driver mutations, aggressiveness, metabolism, neoantigens, and mechanisms of immune evasion. Despite these variables, it is remarkable that certain themes are borne out consistently across the represented samples.

Overall, in this study, we have demonstrated several fairly uniform immune signatures within LNs harboring lung cancer metastases. An influx of neutrophils (or neutrophil-like cells) may represent myeloid derived suppressors, although these were not as well characterized as APOE-high myeloid cells, which we propose as TAMs, given their overt transcriptional reprogramming toward tumorigenesis when compared with noncancerous LN samples. This effect is reflected in CD8 T cell exhaustion, and suggests numerous signaling pathways for directed therapeutics, as well as proxy diagnostic biomarkers for use when biopsy is insufficient to confirm the presence of malignant cells. Given the diversity inherent in tumor biology, it has been reasonably argued that the future of cancer treatment will be personalized, and a routine individual workup may resemble the methodology of our study in the future. At the same time, it is worth considering the emergence of immunotherapy as a new standard of care while being prescribed largely as a one-size-fits-all approach. Our study suggests the development of a broadly applied approach targeting a specific subset of TAMs in lung cancer may yield similar benefits given the engagement of a shared pro-tumorigenic transcriptional program across APOE-high myeloid cells in metastatic LNs. However, future larger-scale studies stratified by lung cancer type will be required to confirm these results, and TAM-targeted therapies remain a long-running work in progress.

## Methods

### Study design and population

This study was conducted in accordance with the Declaration of Helsinki. Study approval was granted by the institutional review boards of the Jesse Brown VA Medical Center and the University of Illinois at Chicago (UIC) Hospital and Health Sciences System. Briefly, subjects aged 18 years or older who were planned to undergo standard-of-care diagnostic bronchoscopy with EBUS to evaluate radiological abnormalities concerning for lung cancer were screened for study enrollment. Subjects were included in the study upon providing informed consent if they did not meet exclusion criteria, which included the use of systemic corticosteroids or other immunosuppressants within 8 weeks and/or a history of respiratory tract infections within 4 weeks prior to the diagnostic procedure, respectively. Study subjects were retrospectively divided into metastatic or non-metastatic (control) groups based on histopathological presence or absence of cancerous cells within sampled intrathoracic LNs. Since all subjects underwent clinically indicated EBUS sampling, additional criteria were established for inclusion into the control group. These included either no evidence of LN disease and no primary lung malignancy with at least six months of follow-up or confirmed negative tumor involvement from surgical LN dissection with no evidence of recurrence for at least one year of follow-up.

### LN needle aspirate

Bronchoscopy and EBUS-TBNA were performed following the standard medical guidelines utilizing an Olympus ViziShot system (Olympus Ltd, Japan) equipped with an ultrasonic 7.5-mHz longitudinal transducer. A 21-gauge needle was used for 3 to 5 needle passes for each LN to establish clinical diagnosis and one or two needle passes were collected for research. The obtained needle aspirates were made into single-cell suspensions. Then, the samples were divided into two aliquots: one processed for single-cell RNA sequencing (scRNA-seq) and the other for mass cytometry (CyTOF).

### Sample preparation

Intrathoracic EBUS-TBNA samples were washed twice in PBS, and tissue was gently digested with Dispase (5 U/ml, STEMCELL Technologies) at 37°C for 30 minutes. Cells were then centrifuged at 300 g for 5 minutes and treated with RBC lysis buffer (Roche) per the manufacturer’s instructions to remove red blood cells, followed by filtration through a 40-μm strainer. The cells were washed twice with PBS and prepared for scRNA-seq and mass cytometry analyses.

### Single-cell RNA Sequencing (scRNA-seq)

Prepared single-cell suspensions with cell viability >80% were used to generate single-cell 3’ transcriptomes targeting 10000 cells/subject. Cells were loaded at a concentration of 1000 cells/µL and captured with barcoded oligo-dT containing gel beads using the Chromium controller (10X Genomics) according to the manufacturer’s instructions. The transcriptomic library was prepared using a 10X Chromium v3 kit (10X Genomics) and sequenced on a Novaseq 6000 instrument using an S4 lane with 2x150nt cycles (University of Illinois Urbana-Champaign DNA Services Lab). Utilizing CellRanger (10X Genomics), raw reads were demultiplexed and aligned against the human reference genome (GRCh38.p14) prior to quantification of single-cell gene expression (UIC Research Informatics Core).

### Quality control of scRNA-seq data

The pre-processed data from each sample was further processed and analyzed using the R package Seurat (v5.1.0)^52^. A Seurat object was created for each sample and subsequently merged into one large Seurat object. Cells with a feature count of less than 300, a percentage of mitochondrial genes greater than 25, or a percentage of mitochondrial genes greater than 2 times the standard deviation were excluded as poor-quality cells. Cells with a feature count greater than 5 times the standard deviation were excluded as doublets or multiplets.

### Batch correction and immune cell selection

To eliminate batch effect, we used the Harmony (version 1.2.0)^53^. The raw data were normalized using the log normalization method. We scaled the normalized data of the top 10,000 variable features, which were selected by the variance stabilizing transformation method. We performed the Principal Component Analysis (PCA) and ran the Harmony. Then, we performed the Uniform Manifold Approximation and Projection (UMAP) and computed the nearest neighbor graph with 30 harmony dimensions. An elbow plot was generated to select the cutoff for the number of dimensions to be utilized. To select immune cell clusters, we applied the method used by Liu et al.^54^. First, we changed the resolution parameter of the ‘FindClusters’ function from 0.1 to 2 by 0.1 to obtain a collection of cell classifications. We then compared the UMAP embedding plot colored by cell type marker genes (T cells: *CCR7, TRABD2A, LEF1, TCF7, IL7R, SELL, CD40LG, TRAC, TCF7, CD3D, CD4, SELL, FOXP3, CTLA4, TIGIT, CD7, CD3E, CD3G, TRBC1*; B cells: *CD79A, IGHM, IGHG3, IGHA2, P2RX5, IGHD, MZB1, CD38, MS4A1, JCHAIN, IGLC1, DERL3*; Myeloid cells: *FCGR3B, CSF3R, CXCR2, FCGR2A, APOC1, C1QA, C1QC, HLA-DPB1, VCAN, THBS1, CX3CR1, LYZ, C1QB, MMP12, CD69, GZMB, LILRA4, LRRC26*; NK cells: *NKG7, GNLY, CD8A, KLRD1*; epithelial cells: *EPCAM, KRT19, KRT18, CHD1*; Fibroblasts: *DCN, THY1, COL1A1, COL1A2*; Endothelial cells: *PECAM1, CLDN5, FLT1, RAMP2*; Neutrophils: *CSF3R, TREM1*; Mast cells: *KIT, MS4A2, GATA2*; Macrophages: *CD68, FCGR1A*; Myeloid dendritic cells (DCs): *ITGAX, ZBTB46, HLA-DRA*; canonical DC 1 (cDC1): *THBD, XCR1, IRF8, CD8A*; canonical DC 2 (cDC2): *CD1C, SIRPA, IRF4*, CD4; plasmacytoid DC (pDC): *IL4R, CLEC4C, NRP1*) with the UMAP embedding plot colored by the clusters output by different resolutions to find the smallest suitable resolution to distinguish cell type markers. In the first round of clustering, we defined immune cells (T cells, B cells, myeloid cells, and NK cells) and selected them for further analysis.

### Immune cell clustering and annotation

Using the immune cell subset identified by the first round of clustering, we redefined the top 10,000 variable features and performed dimension reduction using UMAP with 30 harmony dimensions and again set the cutoff for the number of dimensions utilizing an elbow plot. Cell clusters were defined by comparing the UMAP embedding plots of marker gene expression with UMAP embedding plots of clusters defined with different resolution parameters, as used for immune cell selection. In the first round of clustering, CD4 T cells (*CD3E, CD4, IL7R, CCR7*), CD8 T cells (*CD3E, CD8A, CD8B, NKG7*), Treg cells (*CD3E, CD4, FOXP3, IL2RA, IKZF2*), NK cells (*NCAM1, GNLY, NKG7, GZMB*), Naïve/memory B cells (*CD19, MS4A1, CD79A, CD79B*), Plasma cells (*CD79A, MZB1, JCHAIN, SDC1*), Neutrophils (*FCGR3B, CXCR2, S100A8, CSF3R*), APOE-high myeloid cells (*APOE, CD68, MSR1, C1QA, LYZ*), FNC1-high myeloid cells (*FCN1, FCGR3A, VCAN, S100A8, CCR2*), HLA-DQA1-high myeloid cells *(HLA-DQA1, CD1C, FCER1A, CLEC9A*), pDCs (*CLEC4C, TCF4, LILRA4, IL3RA*), and undetermined lymphocytes were defined and annotated. The cluster expressing more than two cell type markers was subjected to a second round of clustering and assigned to appropriate cell types.

### Differential expression and function enrichment analyses of scRNA-seq data

The differentially expressed genes (DEGs) between control and metastatic LN groups for each cell type were identified using ‘FindMarkers’ function of Seurat utilizing the “MAST” method with 0.1 for min.pct and 0.58 for logfc.threshold parameters. The R packages clusterProfiler (version 4.6.2), msigdbr (version 7.5.1), enrichplot (version 1.18.4) were used to perform gene set enrichment analysis (GSEA) to identify differentially regulated cellular processes and pathways.

### Inference of intercellular communications

Cell-cell interaction of immune cells was inferred using the CellChat R package (version 1.6.1)^40^ for control and metastatic LNs, respectively. The cells annotated as “undetermined lymphocytes” were excluded before running the CellChat. Receptor-ligand interactions inferred by CellChat were prioritized by their interaction strength and differential expression between the two groups.

### scRNA-seq data analysis of the validation cohort in a public domain

The raw UMI matrix, normalized log2 TPM matrix, and cell annotation table of GSE131907 were downloaded and summarized into a Seurat object^45^. Among 55 samples, we selected LN samples for validation (10 surgically resected control LNs and 6 metastatic LNs obtained by EBUS). We then scaled the data, performed PCA, and ran UMAP with 11 principal components. As before, an elbow plot was used to select the cutoff for the number of dimensions. To synchronize the cell subtypes of the validation cohort with our cohort, we checked the expression of marker genes for each cluster and matched them to our cell subtypes.

### Mass cytometry analysis (CyTOF)

For CyTOF analysis, the single-cell suspension was stained as described in our previous publication^55^. Briefly, cells were resuspended in Maxpar Cell Staining Buffer, and an Fc-receptor blocking solution was added for 10 minutes at room temperature. An antibody cocktail was added and incubated for 30 minutes at RT. After washing, a cell intercalation solution was added and incubated for 1 hour at RT, followed by washing with Maxpar Water. Cells were analyzed using Helios™ and opt-SNE from Cytobank (Santa Clara, CA). Metal-conjugated antibodies were purchased from Fluidigm (South San Francisco, CA) and BioLegend (San Diego, CA) and are listed in Supplemental Materials and Methods (Table S3).

### Statistical analysis

The statistical significance of the two groups was assessed by the Mann-Whitney test. The percentage difference between control and metastatic LN groups for each cell type were tested using Chi-squared test. All statistical and computational analyses were performed with the R software environment (version 4.3.2).

## Supplementary information

Supplementary Information XXXXXX

## Data Availability

The single-cell RNA-seq data is available on the NCBI GEO website (GSE277742).

## Code availability

All the codes can be found on GitHub: https://github.com/Minhyung83/Lung_Cancer_LN_immune.
