# Supplementary material for: APOE-high myeloid cells are uniquely associated with metastatic intrathoracic lymph nodes obtained by EBUS-TBNA in primary lung cancer
Source: NPJ Precis Oncol. 2025 Aug 20;9:292. doi: 10.1038/s41698-025-01091-5 (PMC12368159; doi:10.1038/s41698-025-01091-5)
Supplement: Supplementary file 1 — Supplementary Information XXXXXX [file 41698_2025_1091_MOESM1_ESM.pdf]

Table S1

No	Cluster	Freq_Control	Freq_Metastatic	Percent_Control	Percent_Metastatic
1	CD4 T cells	13042	12805	46.66	22.82
2	CD8 T cells	2808	5196	10.05	9.26
3	Treg cells	2536	3980	9.07	7.09
4	NK	1793	2188	6.41	3.90
5	Naive/memory B cells	4348	9673	15.55	17.24
6	Plasma cells	697	3657	2.49	6.52
7	Neutrophils	1084	8560	3.88	15.25
8	APOE high myeloid cells	478	4298	1.71	7.66
9	FCN1 high myeloid cells	521	2195	1.86	3.91
10	HLA-DQA1 high myeloid cells	557	1638	1.99	2.92
11	pDCs	89	493	0.32	0.88
12	undetermined lymphocyte	1	1434	0.00	2.56

Cell number and proportions of major immune cell types identified in scRNA-seq analysis from control and metastatic LNs of EBUS-TBNA cohort.

Table S2

No	Cluster	Freq_Control	Freq_Metastatic	Percent_Control	Percent_Metastatic
1	CD4 T cells	7391	939	49.57	14.62
2	CD8 T cells	1297	605	8.70	9.42
3	Treg cells	784	304	5.26	4.73
4	NK	160	343	1.07	5.34
5	Naive/memory B cells	4618	2042	30.97	31.79
6	Plasma cells	485	145	3.25	2.26
7	APOE high myeloid cells	14	1607	0.09	25.02
8	FCN1 high myeloid cells	14	265	0.09	4.13
9	HLA-DQA1 high myeloid cells	146	152	0.98	2.37
10	pDC	2	21	0.01	0.33

Cell number and proportions of major immune cell types identified in scRNA-seq analysis from control and metastatic LNs of validation cohort

Table S3 Antibody list for CyTOF analysis

Reagents	tag	clone	PN	Source
Anti-Human CD45	89Y	HI30	3089003B	Fluidigm
Purified anti-human CD223 (LAG-3) Antibody*	113Cd	11C3C65	369302	BioLegend
Anti-Human CD19	142Nd	HIB19	3142001B	Fluidigm
Anti-human CD11b	144Nd	ICRF44	3144001B	Fluidigm
Anti-human CD64	146Nd	10.1	3146006B	Fluidigm
Anti-human CD103	151Eu	Ber-ACT8	3151011B	Fluidigm
Anti-human CD66b	152Sm	80H3	3152011B	Fluidigm
Anti-human Tim3	154Sm	F38-2E2	3154010B	Fluidigm
Anti-human CD123	155Gd	6H6	3143014B	Fluidigm
Anti-human CD11c	159Tb	Bu15	3159001B	Fluidigm
Anti-human CD14	160Gd	M5E2	3160001B	Fluidigm
Anti-human CTLA4	161Dy	14D3	3161004B	Fluidigm
Anti-human CD56	163Dy	NCAM16.2	3163007B	Fluidigm
Anti-human CD16	165Ho	B73.1	3165007B	Fluidigm
Purified anti-human CD4 (Maxpar® Ready) Antibody**	166Er	RPA-T4	300541	BioLegend
Anti-human CD206	168Er	15-2	3168008B	Fluidigm
Anti-human CD8	168Er	SK1	3168002B	Fluidigm
Anti-human CD3	170Er	UCHT1	3170001B	Fluidigm
Anti-human HLA-DR	174Yb	L243	3174001B	Fluidigm
Anti-human PD1	175Lu	EH12.2H7	3175008B	Fluidigm
Anti-human TIGIT	209Bi	MBSA43	3209013B	Fluidigm
Iridium			201192A	Fluidigm
* Metal labeled by Maxpar MCP9 Antibody Labeling Kit, 113Cd			201113A	Fluidigm
**Metal labeled by Maxpar® X8 Antibody Labeling Kit, 166Er—4 Rxn			201166A	Fluidigm

Fig. S1

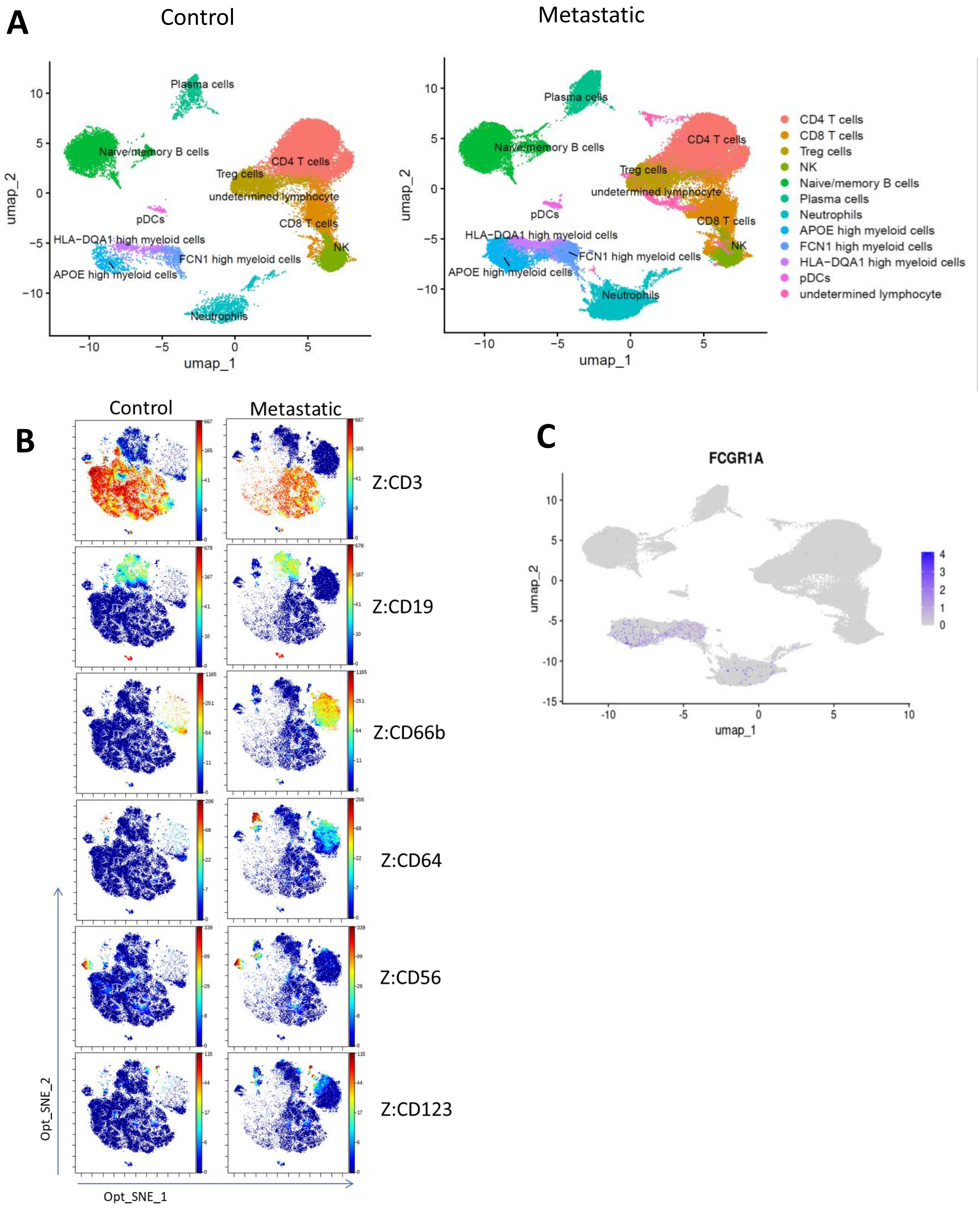

Fig S1.

A) UMAP plots for control LN and metastatic LN groups

B) OPT_SNE plots for control LN and metastatic LN groups with z stack expression of cell type markers, CD3 for T cells, CD19 for B cells, CD66b for Neutrophils, CD64 for myeloid cells, CD56 for NK cells and CD123 for pDC.

C) Feature plot displaying FCGR1A (CD64) expression on combined UMAP plot from control LN and metastatic LN groups

Fig. S2

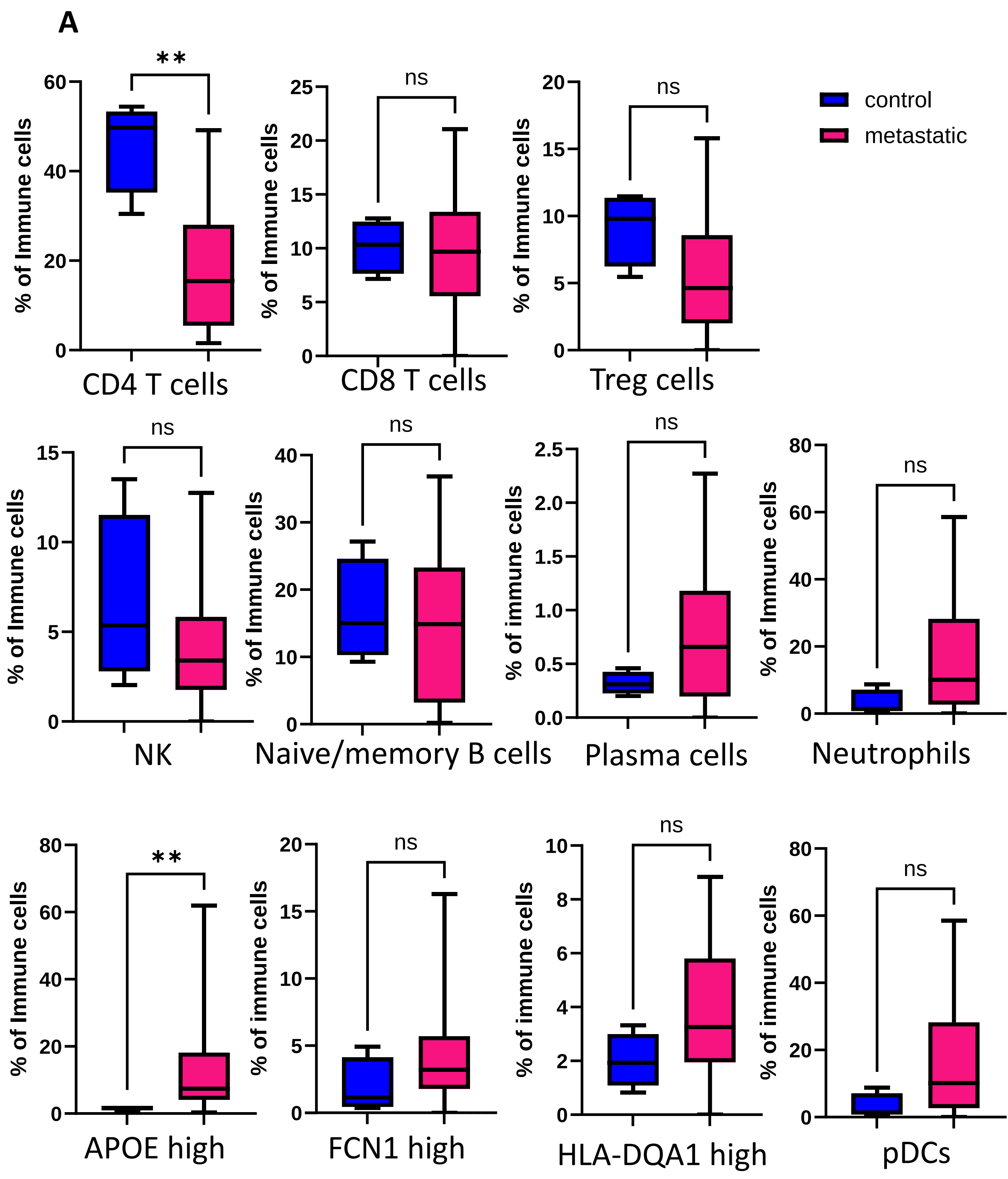
A
control
metastatic
**
ns
% of Immune cells
% of immune cells
CD4 T cells
CD8 T cells
Treg cells
NK
Naive/memory B cells
Plasma cells
Neutrophils
APOE high
FCN1 high
HLA-DQA1 high
pDCs

Fig. S2 continue

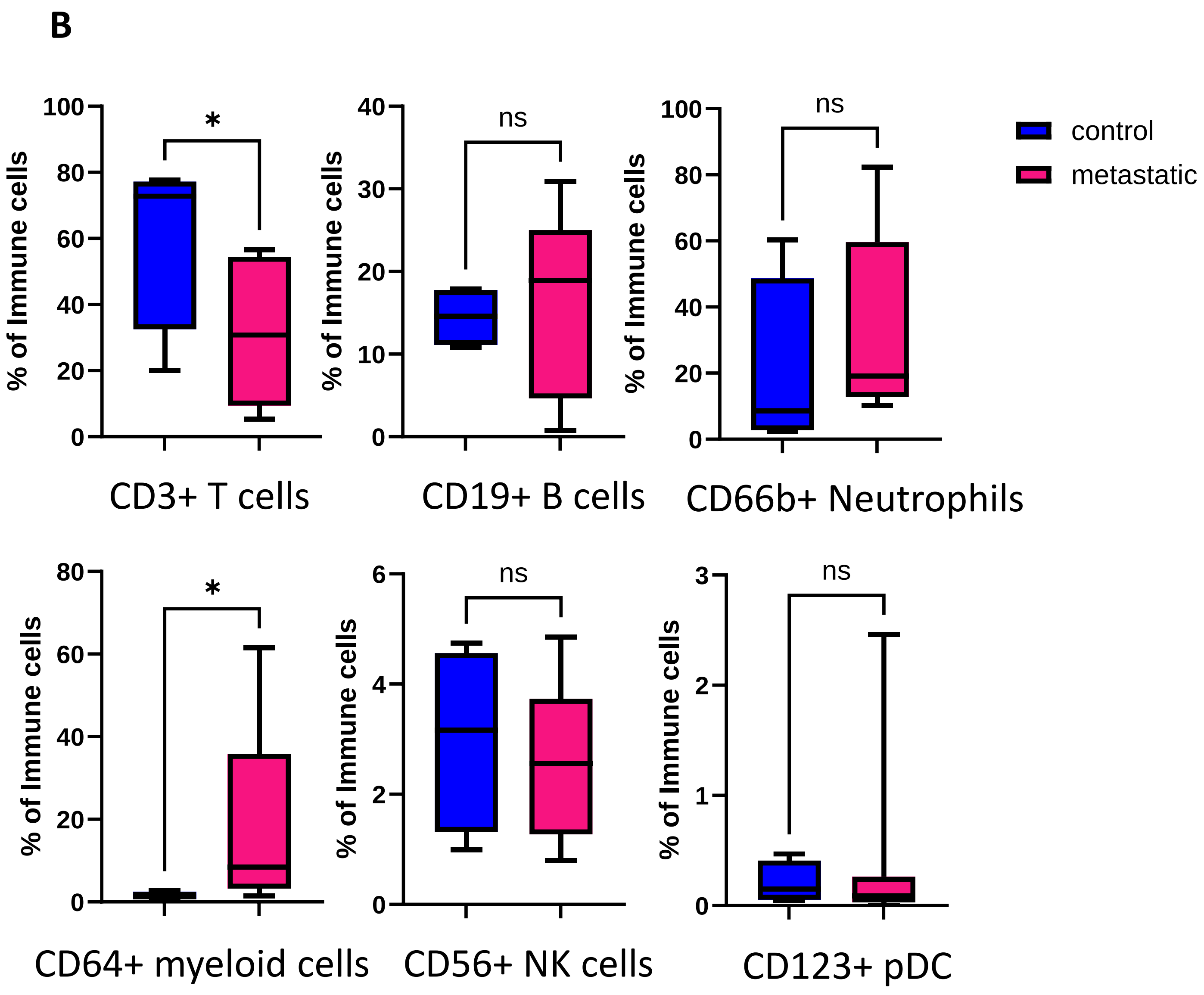

Fig S2.

A) Box plots illustrating the percentage representation of each cell cluster, as identified by scRNA-seq analysis, across individual patients

B) Box plots displaying the percentage representation of each cell types, as identified by CyTOF analysis, across individual patients

Statistical significance was determined by Mann-Whitney test (**: $P < 0.01$, *: $p < 0.05$).

Fig. S3

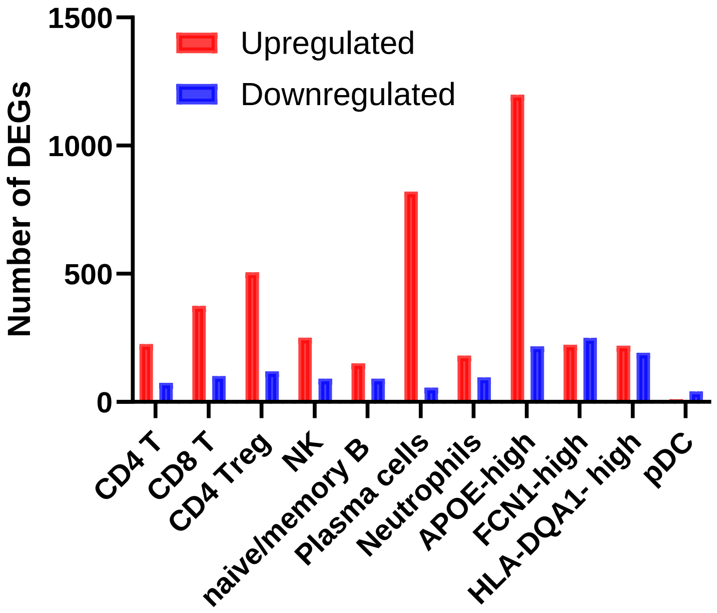

Fig. S3.

Number of Differentially expressed genes (DEGs) count of each cell types in metastatic LN compared to control LN groups.

Fig. S4

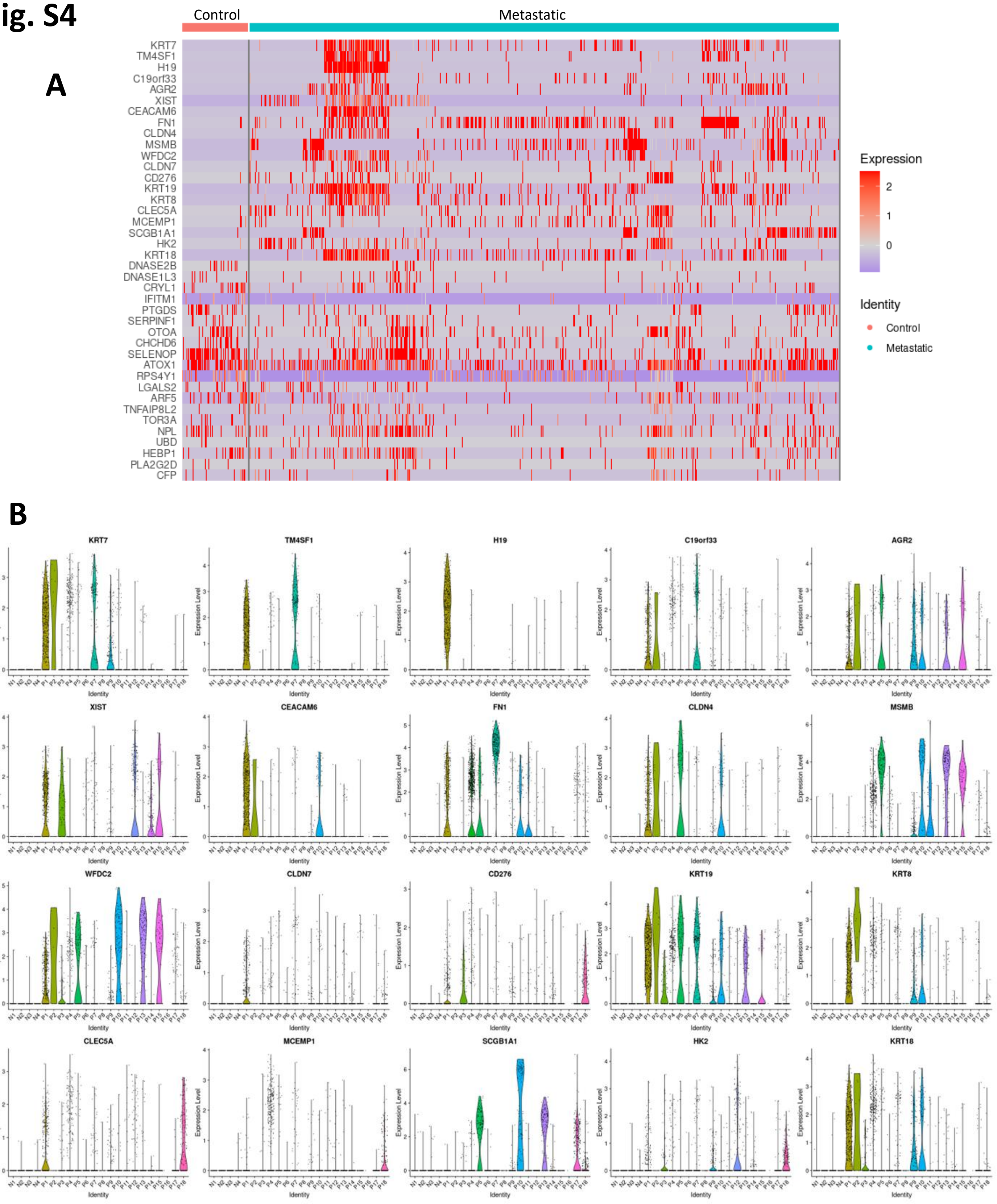

Fig. S4.

A) Heatmap visualizing expression patterns of the 20 upregulated and 20 downregulated DEGs in APOE-high myeloid cell populations from metastatic LN versus control LN.
B) Violin plots depicting the expression levels of the top 20 DEGs in APOE-high myeloid cells across individual patients.

Fig. S5

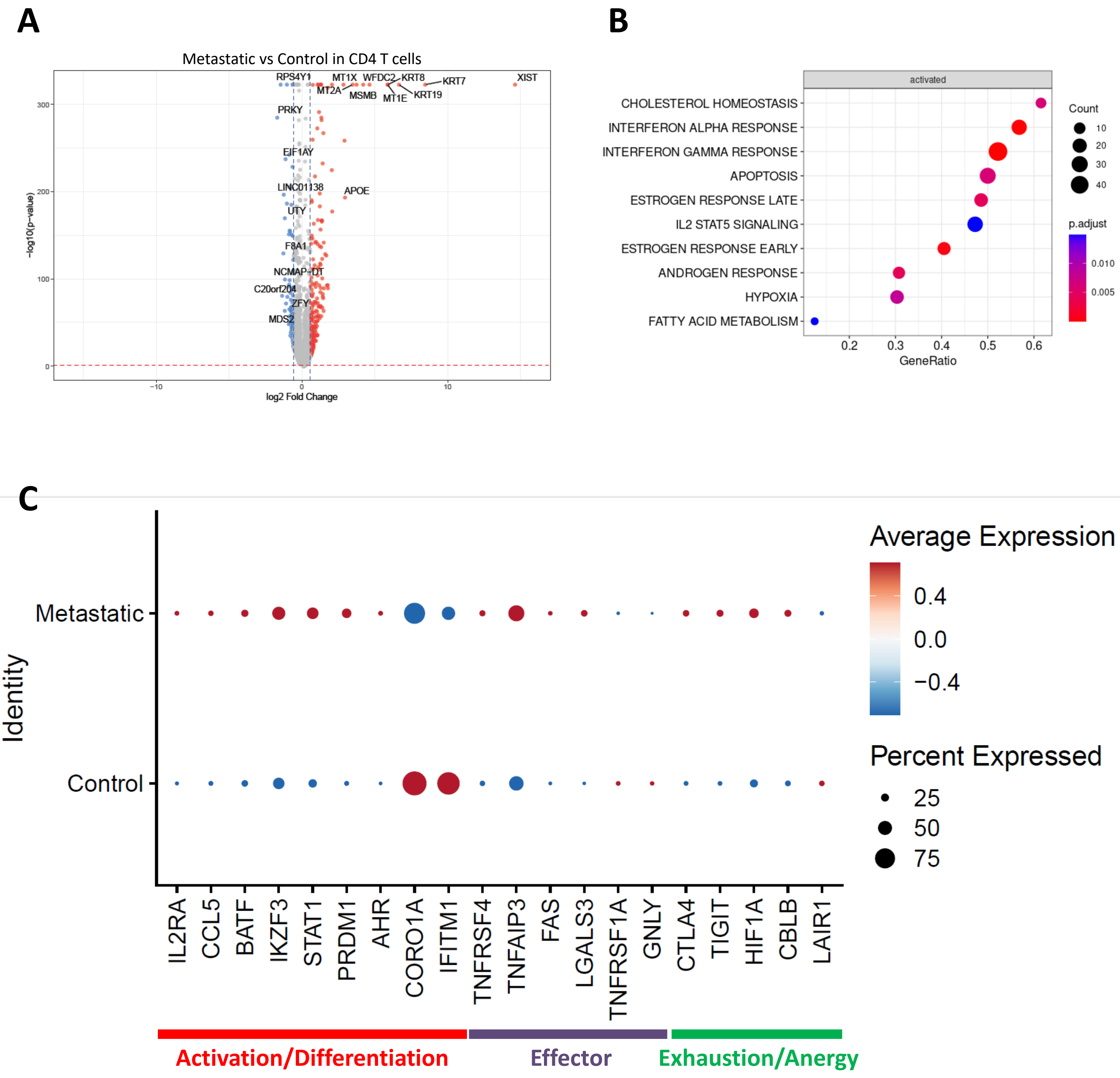

Fig. S5.
Comparative gene expression analysis of CD4 T cells in control LN and metastatic LN groups
A) Volcano plot illustrating gene expression in CD4 T cells of metastatic LN vs control LN groups.
B) Enriched pathways in CD4 T cells of metastatic LN compared to control LN groups.
C) Dot plot showing the expression of selected DEGs categorized by functional groups in CD4 T cells from control and metastatic LN groups.

Fig. S6

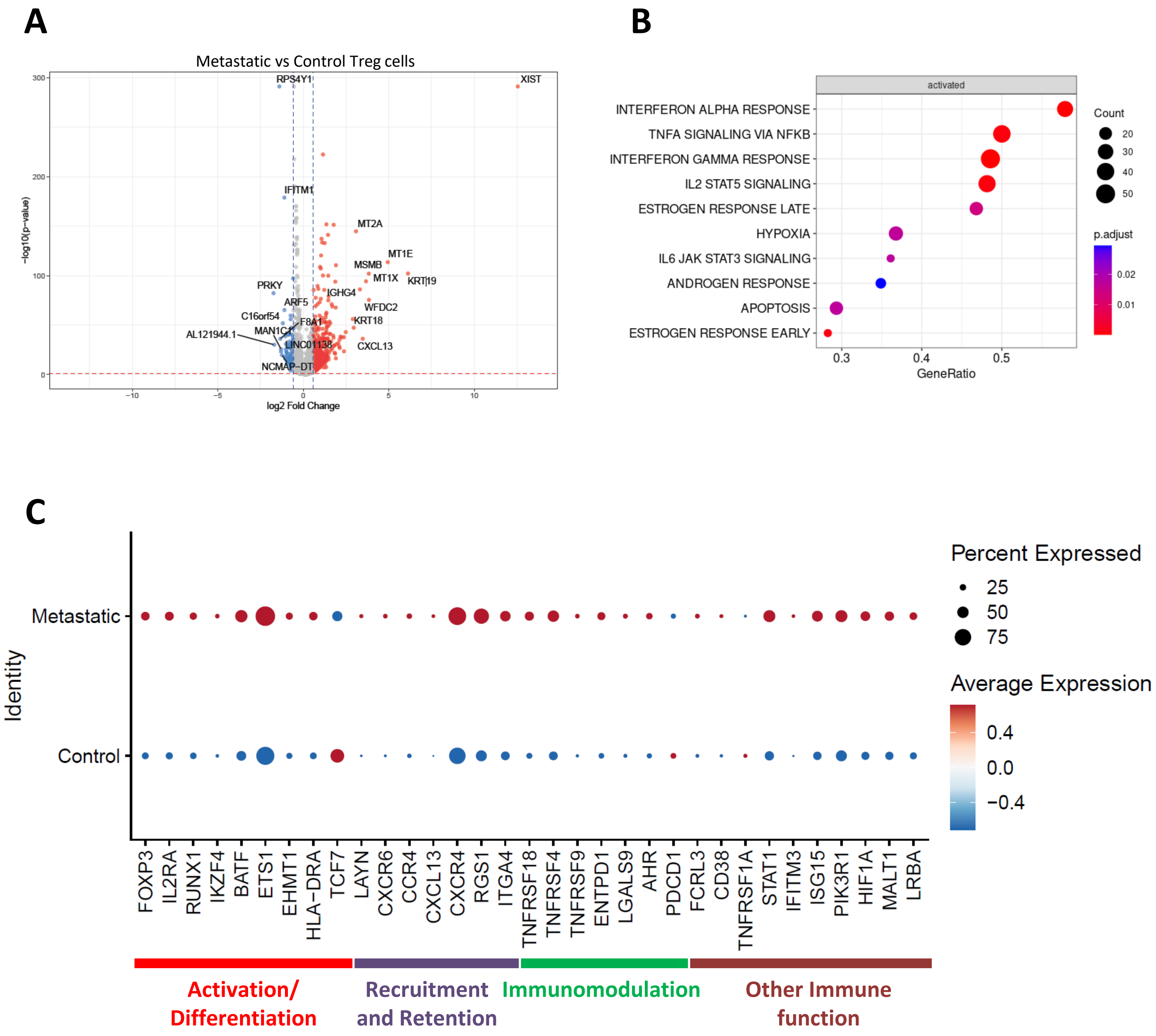

Fig. S6.
Comparative gene expression analysis of Treg cells in control LN and metastatic LN groups
A) Volcano plot illustrating gene expression in Treg cells of metastatic LN vs control LN groups.
B) Enriched pathways in Treg cells of metastatic LN compared to control LN groups.
C) Dot plot showing the expression of selected DEGs categorized by functional groups in Treg cells from control and metastatic LN groups.

Fig. S7

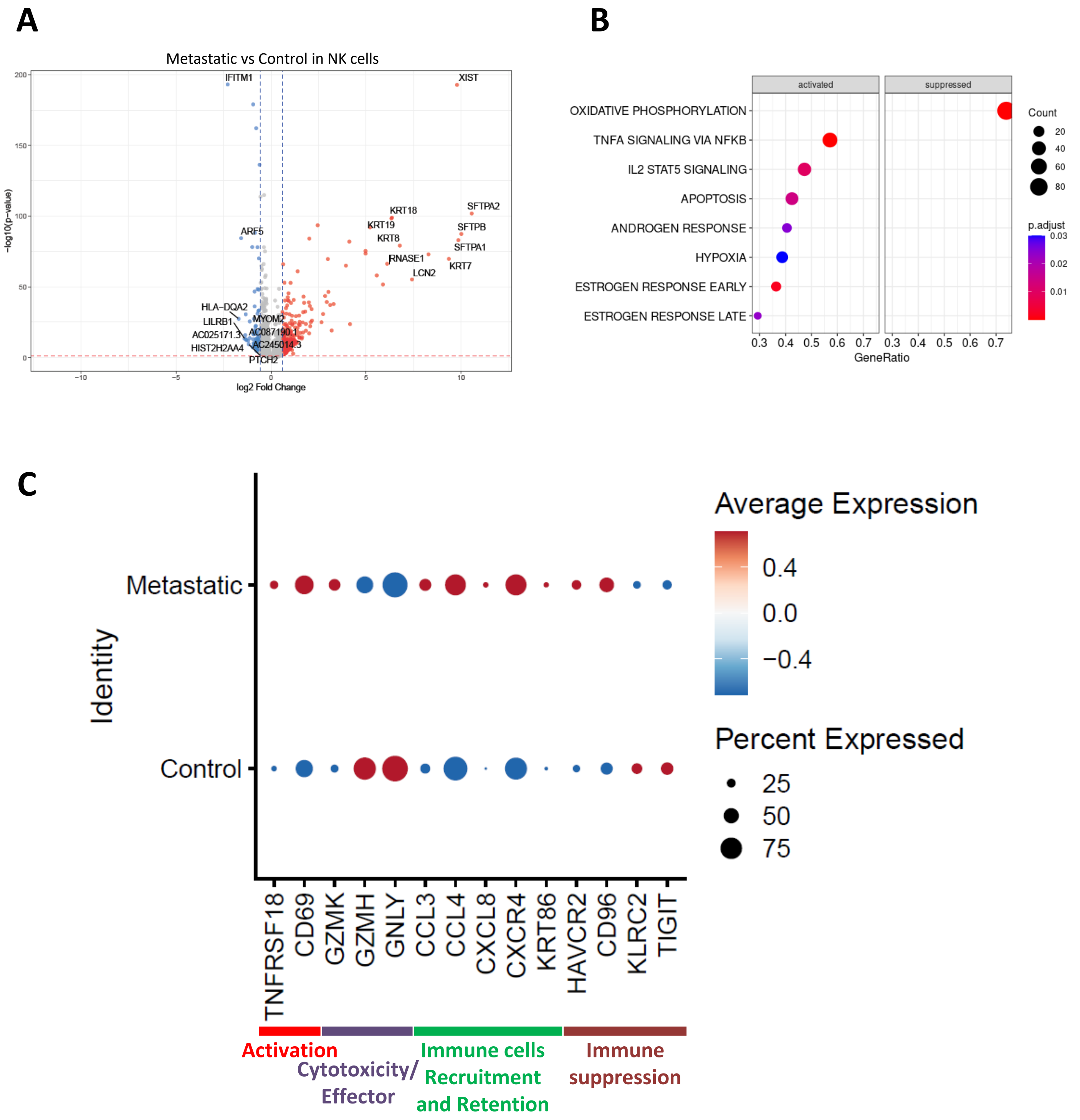

Fig. S7.
Comparative gene expression analysis of NK cells in control LN and metastatic LN groups
A) Volcano plot illustrating gene expression in NK cells of metastatic LN vs control LN groups.
B) Enriched pathways in NK cells of metastatic LN compared to control LN groups.
C) Dot plot showing the expression of selected DEGs categorized by functional groups in NK cells from control and metastatic LN groups.

Fig. S8

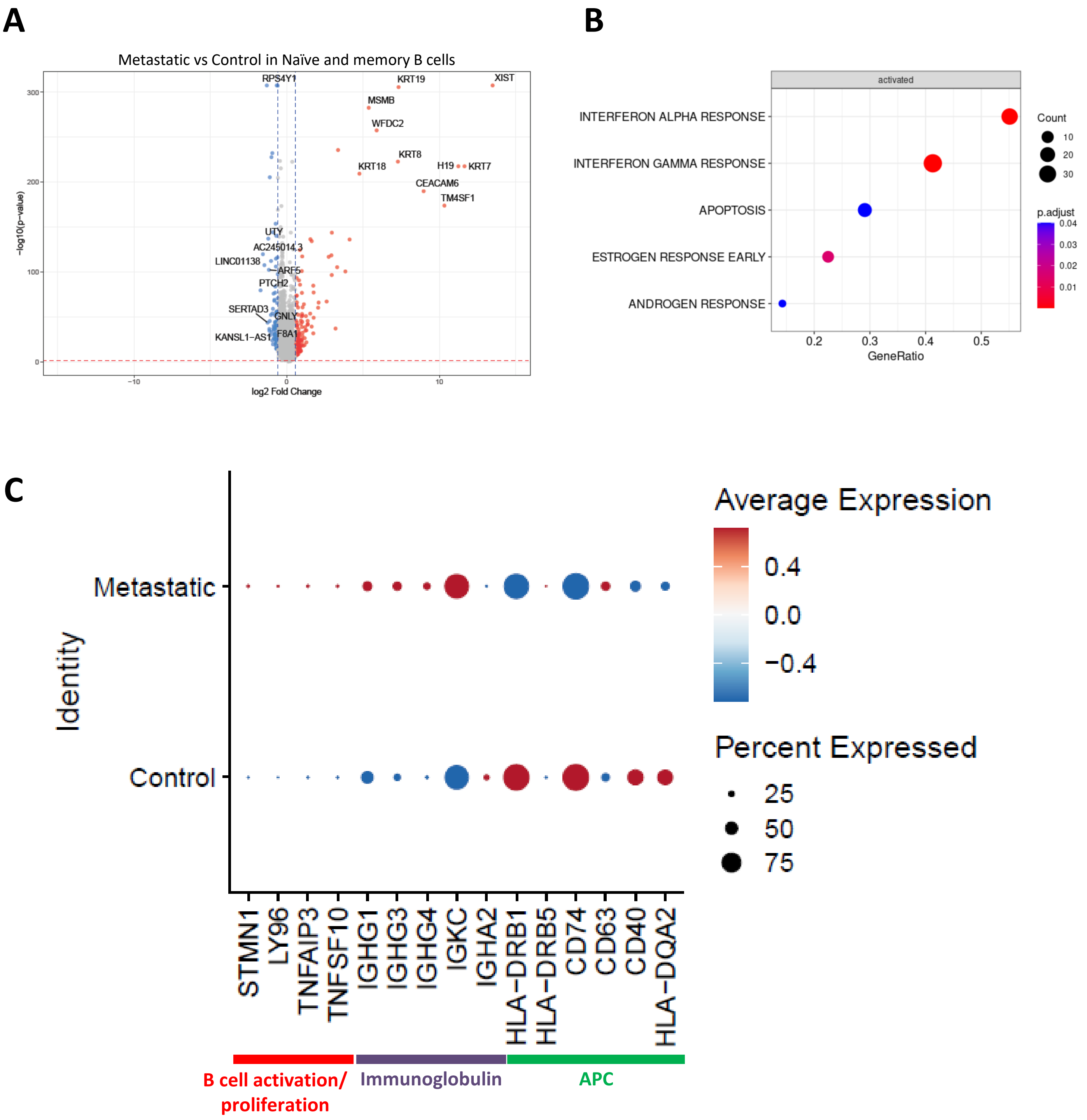

Fig. S8.

Comparative gene expression analysis of Naïve/memory B cells in control LN and metastatic LN groups

A) Volcano plot illustrating gene expression in Naïve/memory B cells of metastatic LN vs control LN groups.

B) Enriched pathways in Naïve/memory B cells of metastatic LN compared to control LN groups.

C) Dot plot showing the expression of selected DEGs categorized by functional groups in Naïve/memory B cells from control and metastatic LN groups.

Fig. S9

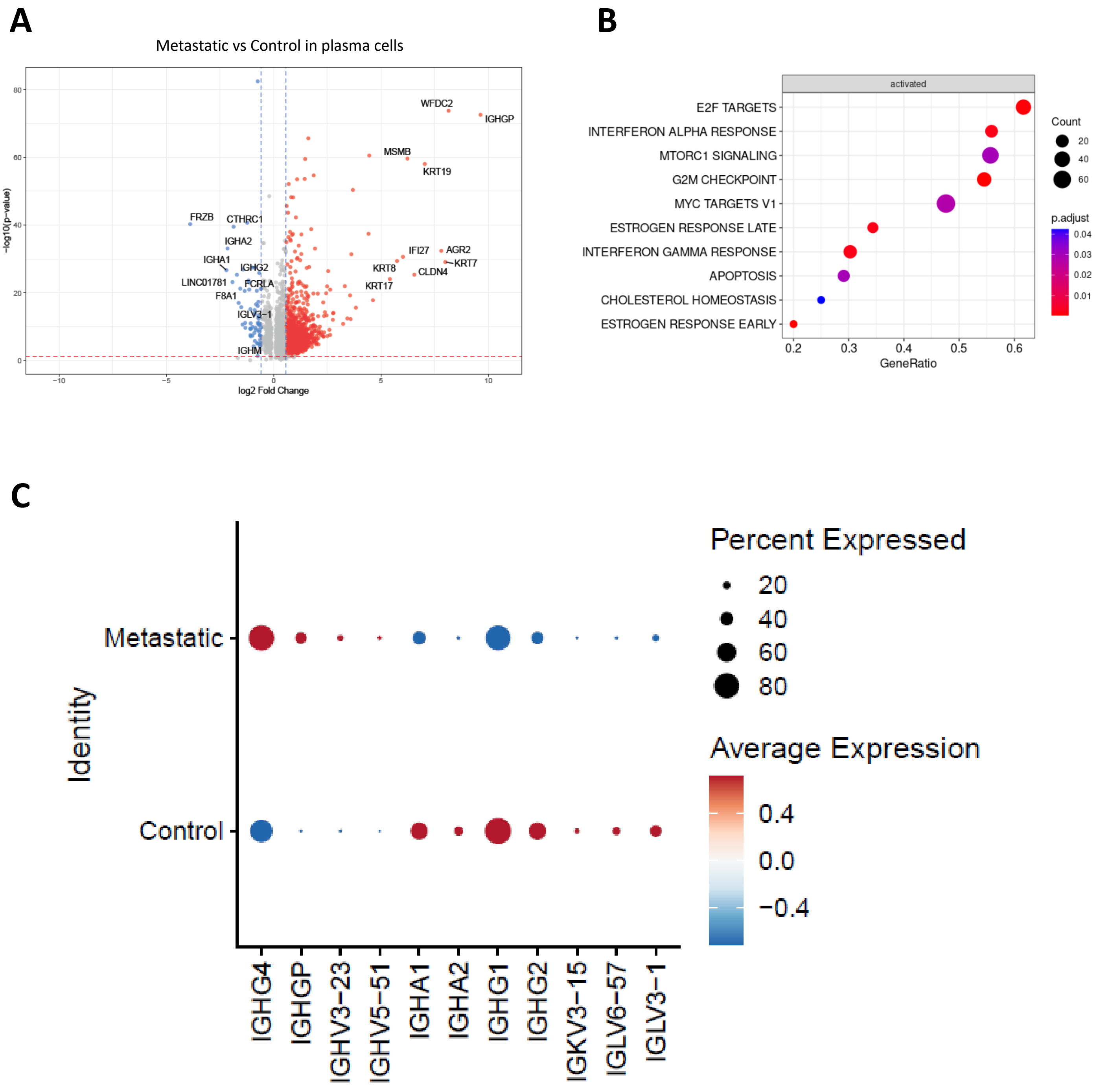

Fig. S9.
Comparative gene expression analysis of plasma cells in control LN and metastatic LN groups
A) Volcano plot illustrating gene expression in plasma cells of metastatic LN vs control LN groups.
B) Enriched pathways in plasma cells of metastatic LN compared to control LN groups.
C) Dot plot showing the expression of selected DEGs categorized by functional groups in plasma cells from control and metastatic LN groups.

Fig. S10

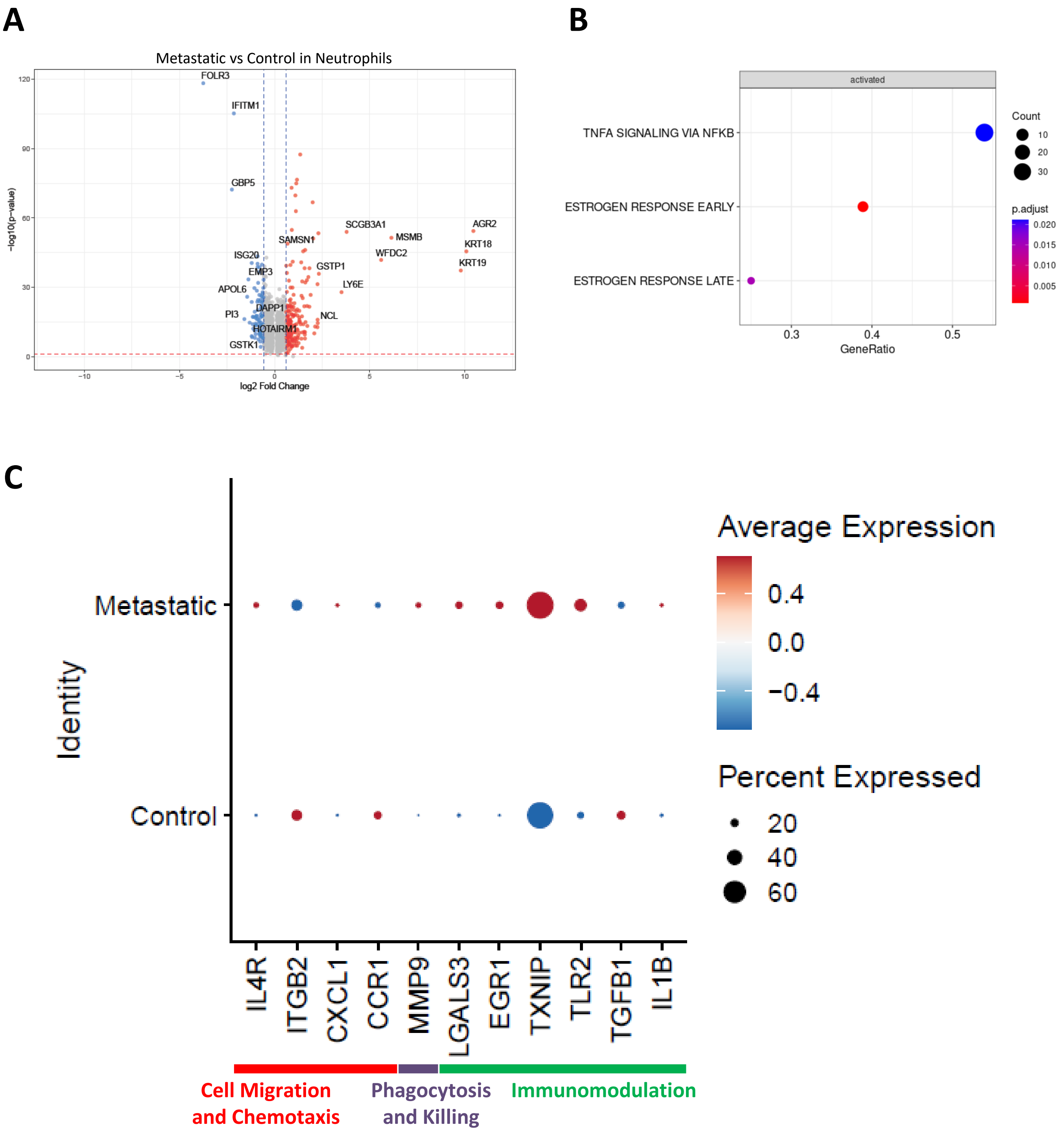

Fig. S10.
Comparative gene expression analysis of neutrophils in control LN and metastatic LN groups
A) Volcano plot illustrating gene expression in neutrophils of metastatic LN vs control LN groups.
B) Enriched pathways in neutrophils of metastatic LN compared to control LN groups.
C) Dot plot showing the expression of selected DEGs categorized by functional groups in neutrophils from control and metastatic LN groups.

Fig. S11

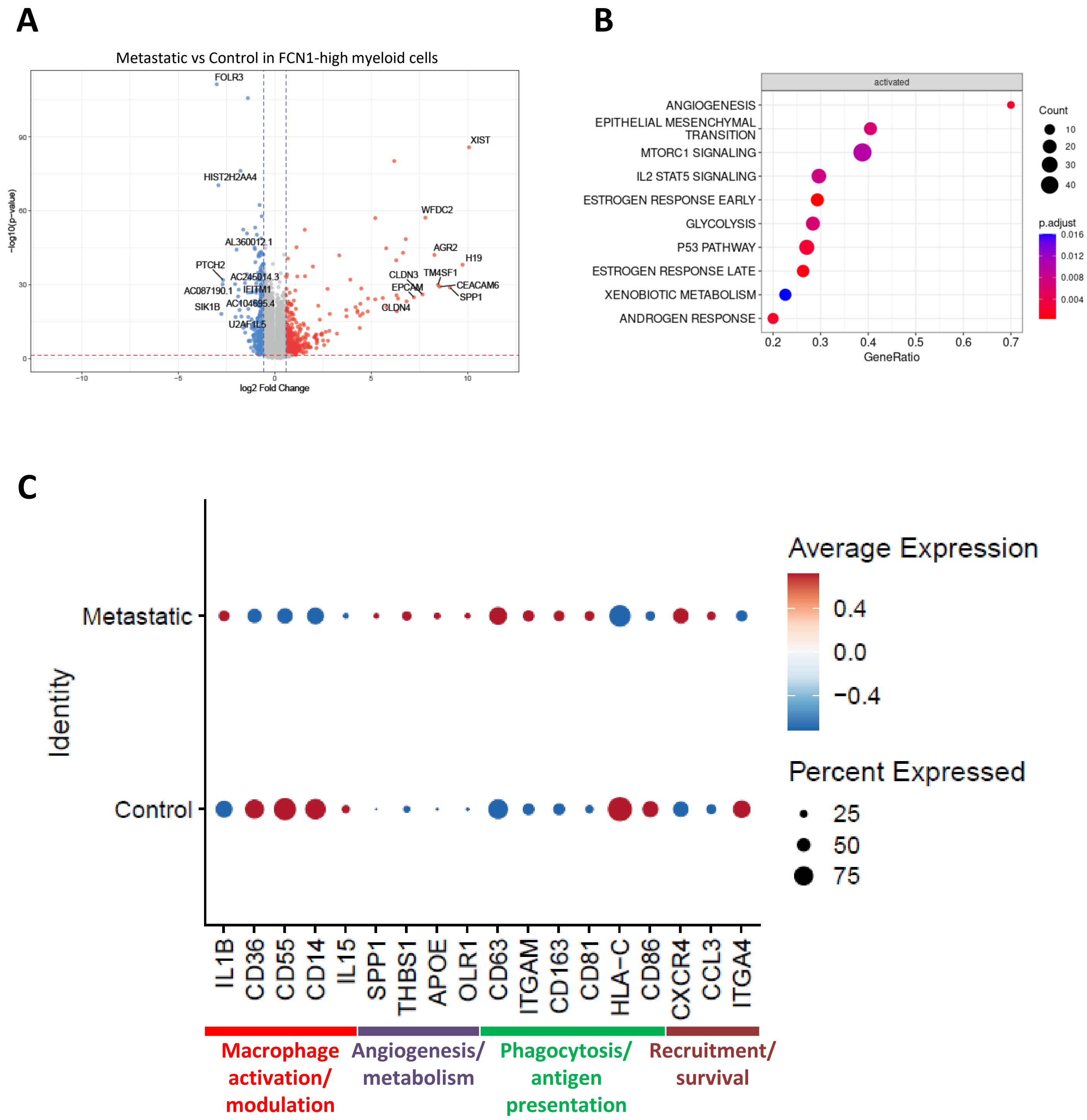

Fig. S11.

Comparative gene expression analysis of FCN1-high myeloid cells in control LN and metastatic LN groups

A) Volcano plot illustrating gene expression in FCN1-high myeloid cells of metastatic LN vs control LN groups.

B) Enriched pathways in FCN1-high myeloid cells of metastatic LN compared to control LN groups.

C) Dot plot showing the expression of selected DEGs categorized by functional groups in FCN1-high myeloid cells from control and metastatic LN groups.

Fig. S12

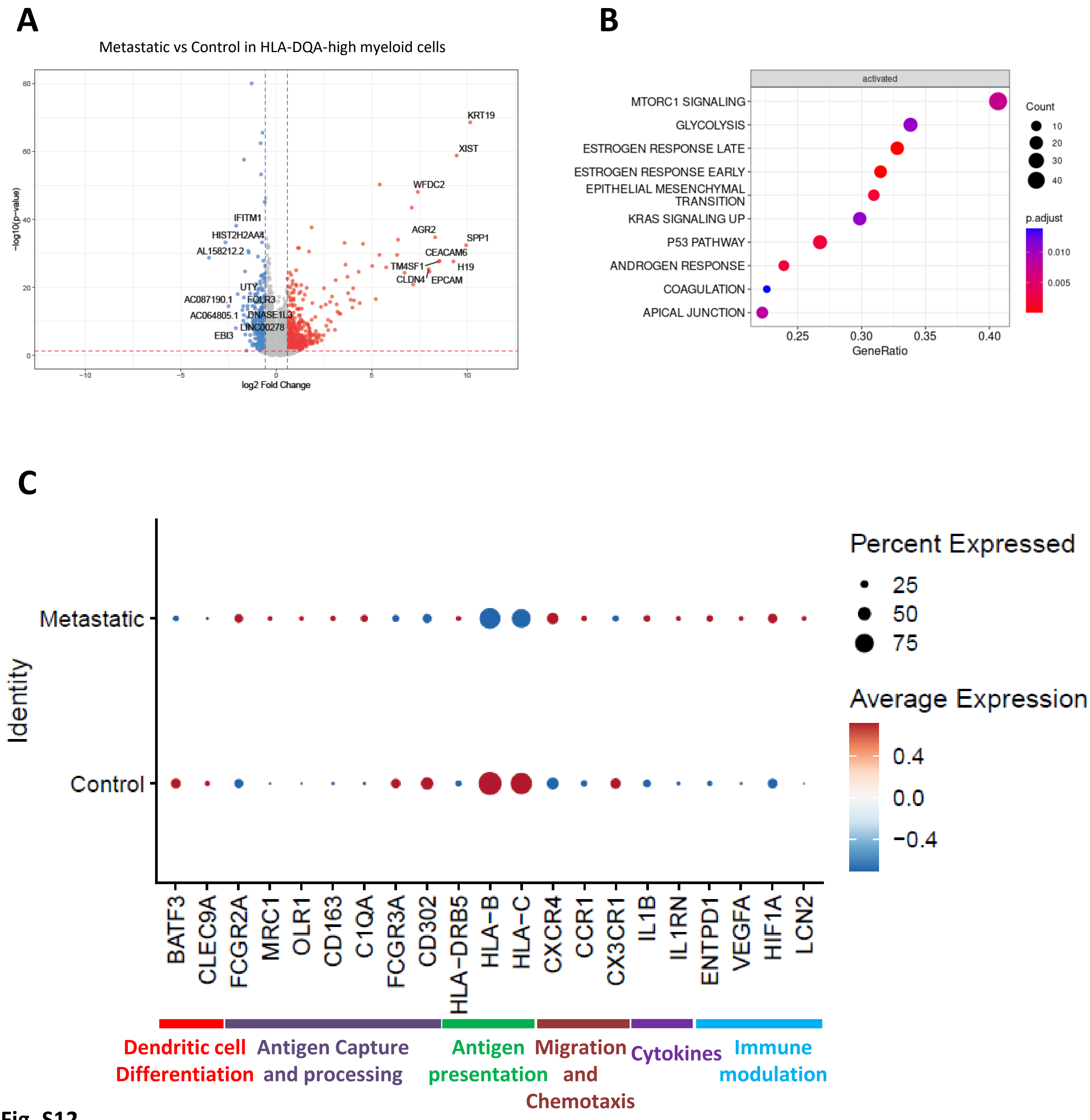

Fig. S12.

Comparative gene expression analysis of HLA-DQA1-high myeloid cells in control LN and metastatic LN groups

A) Volcano plot illustrating gene expression in HLA-DQA1-high myeloid cells of metastatic LN vs control LN groups.

B) Enriched pathways in HLA-DQA1-high myeloid cells of metastatic LN compared to control LN groups.

C) Dot plot showing the expression of selected DEGs categorized by functional groups in HLA-DQA1-high myeloid cells from control and metastatic LN groups.

Fig. S13

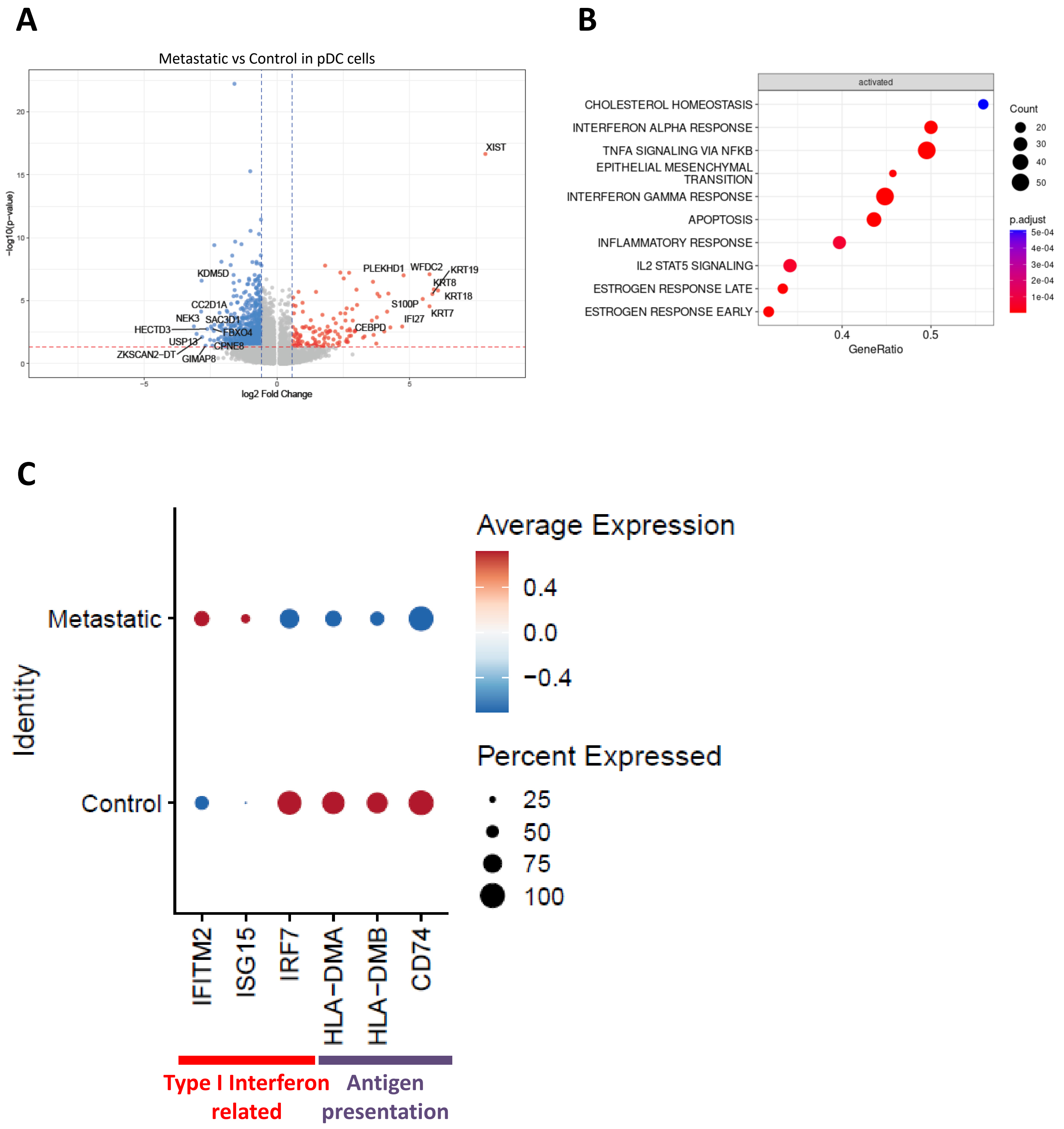

Fig. S13.
Comparative gene expression analysis of pDCs in control LN and metastatic LN groups
A) Volcano plot illustrating gene expression in pDCs of metastatic LN vs control LN groups.
B) Enriched pathways in pDCs of metastatic LN compared to control LN groups.
C) Dot plot showing the expression of selected pDCs categorized by functional groups in Neutrophils from control and metastatic LN groups.

Fig. S14

A **Control**

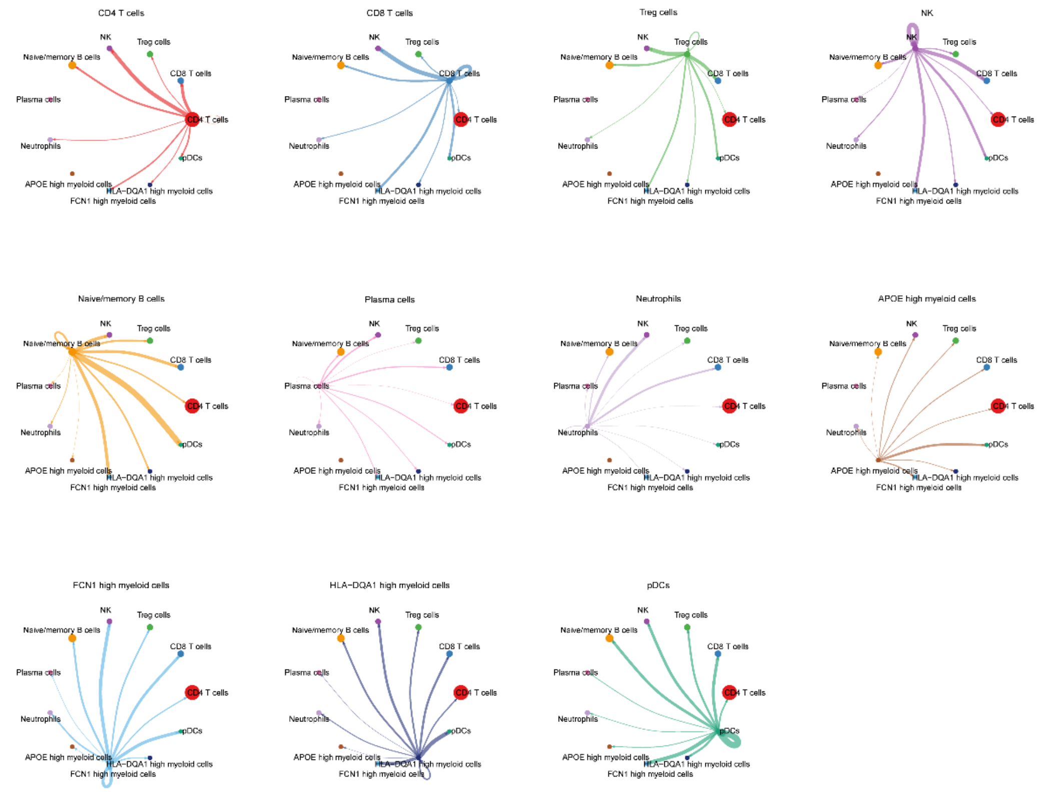

B **Metastatic**

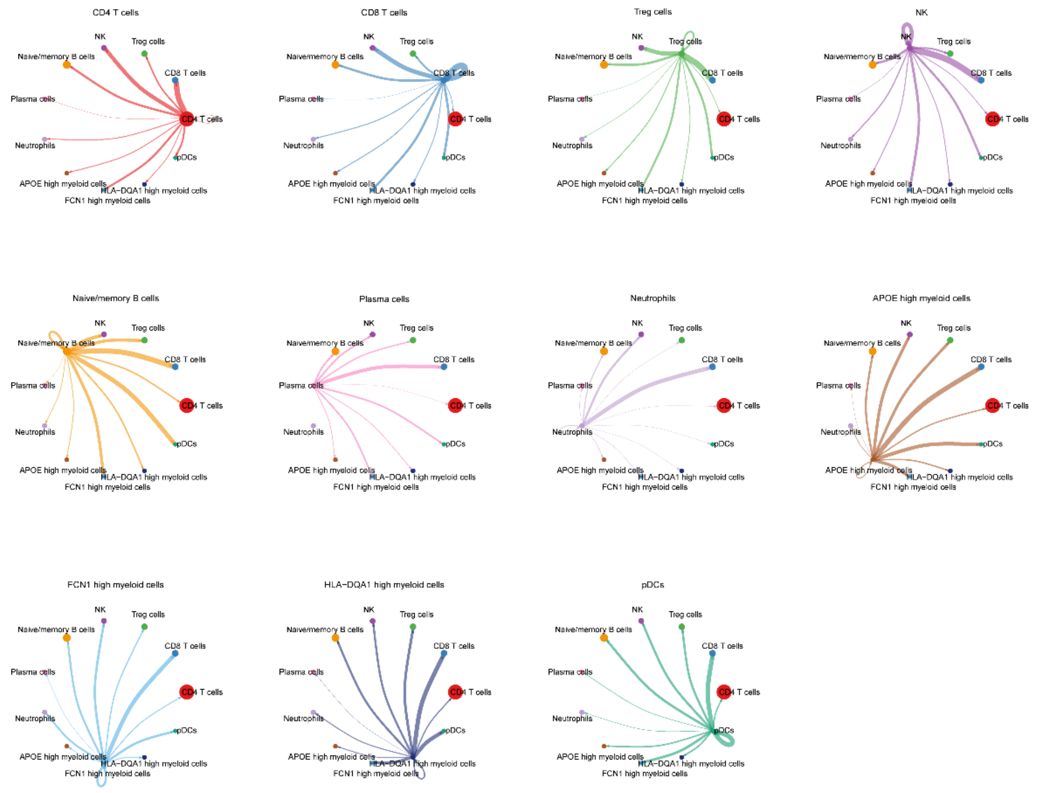

Fig. S14.
Intercellular interactions strength of each cell types in both A) control LN and B) metastatic LN groups. Relative strength was expressed by line thickness

Fig. S15

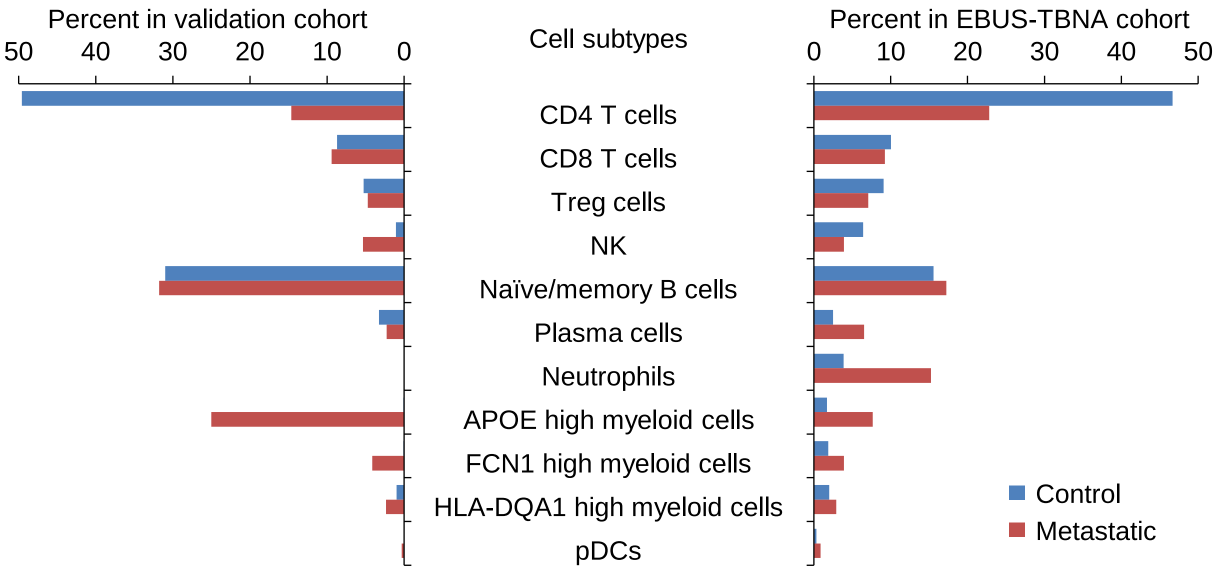

Fig. S15.
Bar plots showing the percentage of cell types in EBUS-TBNA and validation cohorts.
